# ESRRG-PKM2 axis reprograms metabolism to suppress esophageal squamous carcinoma progression and enhance anti-PD-1 therapy efficacy

**DOI:** 10.1186/s12967-023-04347-5

**Published:** 2023-09-07

**Authors:** Tianxiao Wang, Yongjun Zhu, Lu Chen, WenXin Zhang, Huijie Qi, Xiaojin Shi, Mingkang Zhong, Haifei Chen, Qunyi Li

**Affiliations:** 1 Department of Pharmacy, Huashan Hospital, Fudan University, No.12 Urumqi Middle Road, Shanghai, 200040 China; 2 Department of Cardio-Thoracic Surgery, Huashan Hospital, Fudan University, Shanghai, China

**Keywords:** Estrogen-related receptor gamma, Pyruvate kinase M2, Esophageal squamous cell carcinoma, Glycolysis

## Abstract

**Background:**

Glycolysis under normoxic conditions, known as the Warburg effect, confers a selective advantage for the survival and proliferation of many tumors. In this study, we investigated the role of estrogen-related receptor gamma (ESRRG) in metabolic reprogramming in esophageal squamous cell carcinoma (ESCC).

**Methods:**

Bioinformatics analysis indicated that ESRRG expression was decreased in ESCC tissue and associated with poor clinical outcomes. We also examined the effects of altered ESRRG expression on the proliferation and metabolic reprogramming of ESCC cells. We explored the impact of ESRRG on Pyruvate kinase M2 (PKM2) expression and malignant behavior in ESCC.

**Results:**

Our study revealed the inhibitory effects of ESRRG on the growth, tumorigenesis, and glycolysis activity of ESCC cells, which were mediated by the downregulation of PKM2 expression. We further demonstrated that ESRRG directly interacts with the PKM2 promoter to inhibit its activity in ESCC. Notably, the ESRRG-specific agonist, DY131, inhibited ESCC cell proliferation and glycolysis activity by modulating genes in the glycolysis pathway. Moreover, we verified that DY131 exhibits enhanced activity as an immune checkpoint inhibitor, considering the significance of the ESRRG-PKM2 axis in the lactate regulation of ESCC cells.

**Conclusion:**

Our findings provide novel insights into the role of ESRRG-PKM2 signaling in regulating ESCC cell metabolism and immune checkpoint regulation. Additionally, we suggest that DY131 holds promise as a promising therapeutic agent for ESCC treatment.

**Supplementary Information:**

The online version contains supplementary material available at 10.1186/s12967-023-04347-5.

## Introduction

Esophageal carcinoma currently ranks as the seventh most prevalent malignant tumor globally. Global cancer statistics from 2018 reported over 456,000 new esophageal cancer cases and 300,000 deaths, accounting for 3.2% of cancer cases and 5.3% of total deaths [1]. Esophageal squamous cell carcinoma (ESCC) is the predominant histologic subtype of esophageal cancer, with the highest incidence rate in East Asia [2]. Esophagectomy is currently the primary treatment approach for ESCC. However, due to the insidious and aggressive nature of early ESCC symptoms, only a small subset of patients can undergo surgical treatment [3]. Consequently, radiotherapy and chemotherapy are standard treatment approaches for moderate and advanced ESCC patients. However, the overall survival rate remains unsatisfactory [4, 5]. The limited treatment options for ESCC primarily stem from a lack of in-depth understanding of the biology and pathogenesis of this particular cancer type. Therefore, exploring the underlying mechanisms of ESCC pathology is crucial for identifying potential therapeutic targets.

Cancer cell growth is associated with metabolic reprogramming, and is considered one of the hallmarks of cancer [6]. Aerobic glycolysis, a characteristic metabolic reprogramming process, results in higher glycolysis rates in tumor cells than in adjacent tissues, even in the presence of oxygen [7, 8]. Prior research has demonstrated that pyruvate kinase M2 (PKM2), the final rate-limiting enzyme of glycolysis, is predominantly expressed in various cancers, thus providing selective growth advantages over its counterpart, PKM1 [9, 10]. Elevated PKM2 expression enhances glucose uptake, lactate production, and autophagy inhibition, thus promoting oncogenic growth [11]. A recent study found that PKM2 expression in ESCC tissues was significantly upregulated compared to adjacent noncancerous tissues, and high PKM2 expression levels were associated with a poor prognosis in ESCC patients [12].

Estrogen-related receptors (ERRs) are a family of orphan nuclear receptors comprising ESRRA (NR3B1), ESRRB (NR3B2), and ESRRG (NR3B3), which have been implicated in regulating gene expression involved in a wide array of cellular processes [13]. ESRRG was initially identified through yeast two-hybrid screening as interacting with the transcriptional coactivator glucocorticoid receptor interacting protein 1 (GRIP1) [14]. The expression pattern of ESRRG is distinguished by its high levels in fetal and adult human tissues with increased metabolic demands, including the brain, skeletal muscle, placenta, heart and liver [13, 15]. Evidence suggests that ESRRG has a critical role in modulating mitochondrial biogenesis and cellular energy homeostasis [16]. In addition to its importance in metabolic diseases, recent studies have demonstrated that ESRRG expression is correlated with clinical outcomes in various cancer types, drawing increasing attention from oncologists. High expression of ESRRG has been associated with a favorable clinical prognosis in both ovarian and breast cancers [17, 18]. Inhibition of ESRRG expression in breast cancer cells significantly downregulated three carboxylic acid-related genes. It decreased oxygen consumption in tumor cells, increasing extracellular lactate concentration and promoting tumor cell proliferation [19]. Several researchers have proposed that ESRRG may be a potential therapeutic target for gastric and prostate cancers [20, 21]. Considering the strong association between ESRRG expression and cancer prognosis and its crucial role in cell metabolism, ESRRG has emerged as a promising target for cancer diagnosis and therapy.

In this study, we observed a significant reduction in ESRRG expression in ESCC tissues compared to adjacent noncancerous tissues, and found that low levels of ESRRG were associated with a poor prognosis in ESCC patients. The data revealed a direct interaction between ESRRG and the PKM promoter, leading to increased activity in ESCC cells. We also demonstrated that both ESRRG and its specific agonist, DY131, suppressed ESCC cell growth by inhibiting the Warburg effect and enhanced the efficacy of immune checkpoint inhibitors. These findings offer valuable insights into the molecular mechanisms underlying ESCC progression and suggest that activating ESRRG may serve as a potential therapeutic strategy for ESCC patients.

## Materials and methods

### Patients and specimens

This study collected human ESCC tissues and their paired para-carcinoma esophagus tissues from patients at Huashan Hospital, Fudan University, Shanghai, China, from 2016 to 2022. The para-carcinoma esophagus tissues were obtained at least 1 cm away from the tumor border and confirmed to be free of tumor cells by microscopy. To assess the expression level of ESRRG in tumor vs. para-carcinoma tissues and to analyze prognosis, we employed real-time PCR, western blot, and immunohistochemistry (IHC). This study was approved by the Huashan Hospital Ethics Committee, and written informed consent was obtained from all patients.

### Gene expression data analysis

The gene expression data used from the TCGA-ESCC and NCBI GEO databases are publicly available (Accession numbers GSE38129 [22], GSE45670 [23] and GSE53625 [24]). These data were downloaded using BRB array tools for further analysis [25].

### Cell culture

The human esophageal squamous carcinoma cell lines KYSE 150, KYSE 510, KYSE520, ECa109, and TE1, as well as the human esophageal epithelial cell line HECC, were provided by the Chinese Academy of Sciences Cell Bank Type Culture Collection (Shanghai, China). The murine esophageal cancer cell lines AKR were obtained from the ATCC. KYSE 150, KYSE520, ECa109, TE1, and AKR were cultured in RPMI-1640 medium supplemented with 10% fetal bovine serum (FBS, Yeasen Biological Technology Co., Ltd) and 100 U/ml penicillin/streptomycin (Wisent). KYSE 510 was cultured in a mixed medium of 50% F12 (Hyclone) and 50% RPMI-1640. HECC was cultured in high glucose Minimum Essential Medium (MEM, Hyclone) with the same supplements. All of the cell lines were maintained in a CO_2_ incubator at 37 °C with a humidified atmosphere of 5% CO_2_, and 95% air.

### Reagents

DY131 (selective ESRRG agonist, HY-15483), oligomycin (HY-N6782), carbonyl cyanide 4-(trifluoromethoxy) phenylhydrazone (HY-100410), glucose (HY-B0389) and 2-deoxyglucose (HY-13966) were purchased from Med Chem Express (USA) and dissolved in DMSO. These reagents were stored at − 20 °C.

### Virus production and small interfering RNA

The lentiviral expression system was utilized to investigate ESRRG overexpression or knockdown. Lentiviral vectors of ESRRG negative control were obtained from GeneChem Co (Shanghai, China). The ESCC cells (6 × 10^5^) were seeded on six-well plates and infected with the ESRRG or negative control lentiviral vector in 2 mL of culture medium at a multiplicity of infection of 35 plaque-forming units per cell. This was followed by adding 10 times the volume of virus-enhanced infection solution. After an 8-h incubation period, the medium was replaced with 2 mL of fresh medium containing 10% FBS and 1% antibiotic–antimycotic. Stable ESRRG-overexpressing or knockdown cell lines were selected with 1 μg/ml puromycin (Invitrogen, A1113802). The cells were trypsinized after 72 h and prepared for Western blot or qRT-PCR analysis. Similarly, PKM knockdown was determined using the lentiviral expression system. The PKM and negative control vectors were obtained from GeneChem Co (Shanghai, China).

### Cell viability assay

Cell viability was assessed using the Cell Counting Kit-8 (Proteintech Group, Inc, USA) in 96-well plates. Initially, 6 × 10^3^ ESCC cells were seeded per well and incubated under standard culture conditions for 24 h. Following the manufacturer’s instructions, 10 µl of CCK-8 solution was added to each well and incubated for 1 h at 37 °C. The optical density (OD) values at 450 nm were measured using a microplate reader (Biotek, Winooski, VT, USA). Each experiment was conducted three times with five replicates.

### Colony formation assay

To assess cell proliferation, a colony formation assay was performed by plating ESCC cells (1 × 10^3^ cells/well) in 6-well plates and incubating them under standard culture conditions for 7 days. After fixing the cells with 4% paraformaldehyde, 0.1% crystal violet was used to stain the cells for 30 min. The stained colonies were captured, and the number of clones was calculated using ImageJ software.

### EdU assay

To evaluate DNA synthesis, the EdU assay was conducted on ESCC cells seeded in 6-well plates (1 × 10^6^ cells/well) and cultivated overnight. These cells were then incubated with 50 μmol/L EdU (Servicebio, Wuhan, China) for 2 h and stained according to the manufacturer's instructions. Images were captured using a fluorescence microscope (Nikon, Japan), and the percentage of EdU-positive cells was calculated from five random fields in three wells using ImageJ software. DAPI was used to label the nucleus, which appears blue.

### Organoid culture

To obtain patient-derived organoids, single-cell suspensions were prepared by enzymatically digesting fresh ESCC tissues, followed by incubation with 200 U/ml deoxyribonuclease I (Roche, Indianapolis, IN) and collagenase type IV (Sigma, St. Louis, MO) for 1 h at 37 °C. Subsequently, cells were filtered using sterile gauze and 100-mm nylon mesh, then plated in Matrigel (BD) and cultured in DMEM/F12 medium supplemented with 1 × N-2 (Life Technologies, 17,502,048), 1 × B-27 (Life Technologies, 17504044), 1 × Glutamax (Life Technologies), 10 mmol/L HEPES/NaOH (pH 7.4), 50 ng/mL EGF (R&D Systems, 236-EG), 100 ng/mL Noggin (R&D Systems, 1967-NG), 100 ng/mL R-Spondin 1 (R&D Systems, 7150-RS), 100 ng/mL FGF10 (PeproTech, 100-26), and 10 mmol/L Y27632 (WAKO, 253-00513). Approximately 2000 cells were plated per well on day 1 for organoid formation assays, and the number and diameter sizes of the organoids were detected and compared on day 14.

### Measurement of glucose uptake, lactate production, pyruvate and ATP content

ESCC cells (1 × 10^6^) were seeded into a 6-well plate and cultured for 24 h until cells adhered. ATP and pyruvate levels were assessed as per the ATP Assay kit and Pyruvate Assay kit instructions (Sangon Biotech, Shangahi China). To measure glucose and lactate concentrations, the culture medium was swapped with phenol-red free DMEM containing 1% FBS for another 24 h, after which the medium was collected. Glucose uptake was evaluated using a kit (Abcam, USA), while the consumed glucose level was determined by subtracting the detected glucose concentration in the medium from the initial one. The lactate production assessment followed the Lactate Assay kit instructions (Sangon Biotech, Shanghai, China). The obtained results were adjusted based on cell numbers to ensure accuracy.

### Assays of ECAR and OCR

We seeded 10,000 cells per well in 96-well plates and treated them according to the instructions. Following that, cells were treated with extracellular acidification rate (ECAR) reagents as directed by the manufacturer (ab197244, Abcam, UK). Micro-plate readers (Victor, 3 PerkinElmer) collected ECAR signals every 5 min for 120 min using 380 nm excitation and 615 nm emission wavelengths, respectively. Then, the oxygen consumption rate (OCR) was measured using a Seahorse XF24 Analyzer (Seahorse). An equilibration medium containing 25 mM glucose, 1 mM pyruvate, and 2 mM glutamine was used to equilibrate cells in a bicarbonate-free DMEM medium. All measurements were done in 3 wells per condition per experiment and repeated at least thrice [26].

### Western blot analysis

Western blotting was performed following previously described methods. ESRRG (Proteintech, Cat No. 14017-1-AP), PKM2 (Cell Signaling Technology, Cat No. 4053), β-actin (Cell Signaling Technology, Cat No. 4970), CD8 (Cell Signaling Technology, Cat No. 85336), and GAPDH (Santa Cruz Biotechnology, Cat No. sc47724) were used as primary antibodies. The primary antibodies were followed by appropriate secondary antibodies conjugated to horseradish peroxidase. Antibody-protein complexes were detected using an enhanced chemiluminescence (ECL) immunoblotting detection reagent. The signals were analyzed using a LAS-3000 image analyzer and MultiGauge software (Fuji Film). Densitometric analysis of Western blot results was performed with ImageJ software.

### Quantitative real‑time polymerase chain reaction

Total RNA was extracted from cells using the Trizol reagent and Ultrapure RNA kit (CW Biotech, China) according to the manufacturer’s instructions. The Superscript™ reverse transcription system (Takara, Dalian, Japan) was used to reverse-transcribe 2 μg of total RNA into cDNA. The ABI 7500 Real-Time PCR system (Applied Biosystems, Foster City, CA, USA) was employed to perform quantitative real-time reverse transcription polymerase chain reaction (RT-PCR) reactions using SYBR Green PCR master mix reagents (Takara, Dalian, Japan). The primer sequences were selected from the RTPrimerDB database (http://medgen.ugent.be/rtprimerdb/). The relative quantification of the target gene was calculated using the 2-ΔΔCt method, normalized to β-actin gene expression.

### RNA-Seq and data analysis

Total RNA was extracted from TE1 cells with ESRRG overexpression and control cells using Trizol reagent (Invitrogen, Carlsbad) following the manufacturer’s protocol. The quality and quantity of RNA were assessed using a NanoDrop ND-1000 spectrophotometer. The cDNA library was prepared using the MGI Stranded RNA-Seq Library Preparation Kit according to the manufacturer's instructions. RNA sequencing was performed on an MGI MGISEQ-2000 system following the manufacturer's guidelines. To gain further insight into the biological pathways involved in ESCC related to ESRRG, gene set enrichment analysis (GSEA) was conducted.

### Dual-luciferase assays

Dual-luciferase assays were performed to evaluate the activity of the − 1900 to + 50 regions of the PKM promoter. Cells were transfected with plasmids containing a single fragment in the region to control luciferase expression and a Renilla luciferase reporter for 24 h. The firefly and Renilla luciferase activity levels were measured using the Dual-Luciferase Reporter Assay System (Promega).

### Histopathology and immunohistochemistry

To evaluate ESRRG expression in patient tissue sections, the sections were subjected to antigen retrieval by heating in a microwave oven at 100 °C for 15 min using citrate buffer (pH 6.0) after deparaffinization and rehydration. The sections were then incubated overnight at 4 °C with an ESRRG antibody (1:200) or PKM2 antibody (1:500). Subsequently, a secondary antibody (goat anti-rabbit conjugated to horseradish peroxidase, 1:200; #ab97051, Abcam) was applied to the sections for 1 h at room temperature. The sections were stained using 3,3′-diaminobenzidine tetrahydrochloride (Longisland, Shanghai, China), and the expression of ESRRG was assessed based on staining intensity and extent. Staining intensity was scored as 0 (negative), 1 (weak), 2 (moderate), or 3 (strong), while extent was scored based on the percentage of positively stained cells: 0 (0%), 1 (1–25%), 2 (26–50%), or 3 (51–75%). The expression score was calculated by multiplying the intensity and extent scores. Immunohistological analysis was performed using paraffin-embedded sections according to the manufacturer's instructions to assess anti-PKM2 and anti-CD8 expression for tumor tissues obtained from mice-bearing tumors. Subsequently, major organs were subjected to histological examination using hematoxylin/eosin (HE) staining. Mice were euthanized at the end of the treatment prior to the examination.

### Single-cell preparation from tumor tissue and flow cytometry analysis

Following the sacrifice of mice, subcutaneous tumor tissues were immediately minced and processed for single-cell analysis using the tumor dissociation kit following the manufacturer’s instructions (Miltenyi Biotec). The minced tissues underwent an initial filtration step, and red blood cell lysis before tumor-infiltrating leukocytes were isolated using gradient centrifugation with Percoll (GE Healthcare). Single-cell suspensions were prepared, and Fc block (BioLegend) was used for blocking. Flow cytometry analysis was performed, and immune populations were stained with antibodies, including CD45, CD3, CD4, CD8, and NK-1.1. Additionally, intracellular markers such as Foxp3 and Granzyme B were stained, and intracellular cytokine levels were assessed after incubation with Cell Stimulation Cocktail (Invitrogen) and staining for IFN-γ following fixation and permeabilization. The gating strategy for flow cytometry analysis of lymphoid and myeloid populations in AKR tumors is shown in Additional file 1: Fig. S9.

### Esophageal squamous carcinoma xenografts

To establish human esophageal squamous carcinoma cell xenografts, 2 × 10^6^ ESCC cells suspended in PBS/Matrigel were injected subcutaneously into the right flank region of 6-week-old BALB/c nude mice. Tumor growth was monitored every 5 days until the subcutaneous tumors reached an average size of over 100 mm^3^. The mice were weighed every 5 days to monitor their general health. In pharmacological experiments, mice were intraperitoneally treated with either DY131 (40 or 80 mg/kg) or vehicle (1% DMSO + ddH_2_O) every 2 days for 25 days, starting on day 5 after tumor injection. For anti-programmed death 1 (PD1) treatments, mice were intraperitoneally administered mouse anti-PD1 antibodies (100 µg per mouse) or IgG isotype control (BioXCell) every 3 days for 25 days, beginning on day 5 after tumor injection. All animal procedures were conducted following the approved protocols of Fudan University’s Institutional Animal Care and Use Committee. Efforts were made to minimize the animals’ distress, pain, or discomfort throughout the study.

### Statistical analysis

Statistical analysis was performed using GraphPad Prism 7.0 and SPSS 16.0 software.

The Wilcoxon test was employed to assess the differences between two groups in the context of bioinformatics data analysis. Group differences in both in vitro and in vivo experiments were analyzed using a one-way analysis of variance (ANOVA) followed by Dunnett’s post hoc test. Prior to analysis, the normality of data distribution was assessed using the Kolmogorov–Smirnov test, and the homogeneity of variances was assessed using Levene’s test. The results indicated that all data from different groups satisfied the normality and variance homogeneity assumptions. Data are presented as mean ± SD. A p-value less than 0.05 was considered statistically significant.

## Results

### ESRRG was expressed at low levels in ESCC tissues and was associated with a poor prognosis

**Fig. 1:**
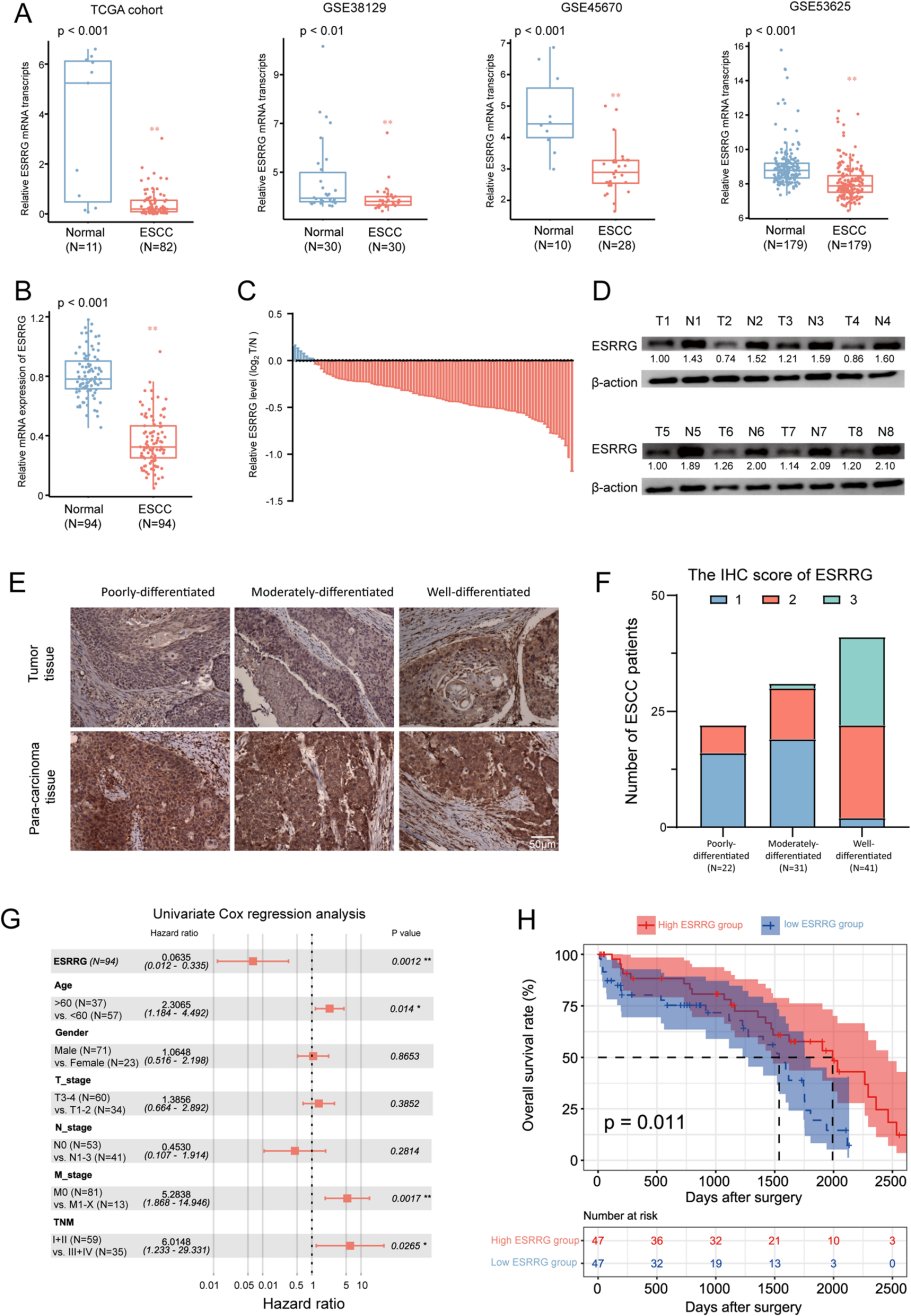
Downregulation of ESRRG and prognostic significance in ESCC. **A** ESRRG expression in paired samples of tumor tissues and nontumorous adjacent normal esophagus tissues from patients with esophageal squamous cell carcinoma (ESCC) in the TCGA and three GSE cohorts. **B**, **C** mRNA levels of ESRRG were evaluated using quantitative real-time PCR in 94 paired samples from patients with ESCC. **D** 8 paired samples from patients with ESCC were analyzed by Western blot. **E** Immunohistochemical staining for ESRRG was performed from patients with ESCC. **F** The correlation between the IHC score of ESRRG and the level of tumor cell differentiation in 94 ESCC tissues. **G** Univariable analyses were conducted in the ESCC cohort, where all bars were corresponded to 95% CIs. H Kaplan–Meier analysis for OS was performed according to ESRRG mRNA levels. *P < 0.05 or **P < 0.01 indicates significant differences from the normal group as assessed by Wilcox test

To investigate the role of ESRRG in esophageal squamous cell carcinoma (ESCC), we analyzed its expression in various datasets, including TCGA-ESCC, GSE38129, and GSE53625. Our analysis revealed a consistent downregulation of ESRRG expression in ESCC tissues compared to adjacent tissues (Fig. 1A). In contrast, the expression of other members of the ERR family (ESRRA and ESRRB) did not show a consistent trend in tumor and adjacent tissues (Additional file 1: Fig. S1).

We further validated the decreased expression of ESRRG in ESCC by analyzing 94 pairs of tumors and paracancerous tissues from ESCC patients using real-time PCR. The results showed that 92.5% of tumor tissues exhibited decreased mRNA levels of ESRRG compared to paracancerous tissues, while only 7.5% of tumor tissues showed increased ESRRG expression (Fig. 1B, C). Western blot analysis (Fig. 1D) confirmed these findings at the protein level. Next, we conducted an analysis to examine the potential associations between the IHC score of ESRRG and the level of tumor cell differentiation in a sample of 94 patients with ESCC. The findings of our investigation revealed significant negative correlation between the IHC score of ESRRG and the degree of tumor differentiation among the patients (Fig. 1E, F).

We conducted further analysis to determine the clinical significance of ESRRG downregulation in ESCC patients. Univariate Cox analysis incorporating ESRRG expression, age, gender, pathological stage, and tumor grade revealed that ESRRG is an independent predictive marker for the prognosis of ESCC (HR = 0.064, 95% CI 0.012−0.335, P < 0.01, Fig. 1G). Kaplan–Meier analysis demonstrated that ESCC patients with lower ESRRG expression had worse overall survival (OS) compared to patients with higher ESRRG levels (Fig. 1H). These findings suggest that ESRRG may serve as a potential clinical biomarker for predicting disease outcome in ESCC patients.

### ESRRG suppresses proliferation of ESCC cells in vitro and in vivo

To investigate the biological function of ESRRG in ESCC cells, we first examined the mRNA and protein expression levels of ESRRG in different ESCC cell lines (Additional file 1: Fig. S2A, B). The findings indicate that the mRNA and protein expression levels of ESRRG were significantly elevated in normal esophageal epithelial cells when compared to ESCC cells. Moreover, the expression of ESRRG in ECA109 and KYSE510 cell lines was found to be higher than that observed in TE1 and KYSE150 cell lines among the five ESCC lines examined. Based on these results, we established stable models of ESRRG knockdown in KYSE 510 and ECa109 cell lines, as well as stable models of ESRRG overexpression in TE1 and KYSE150 cell lines. The efficiency of ESRRG overexpression or knockdown in ESCC cell lines was confirmed by real-time PCR and Western blot analysis (Additional file 1: Fig. S3).

**Fig. 2:**
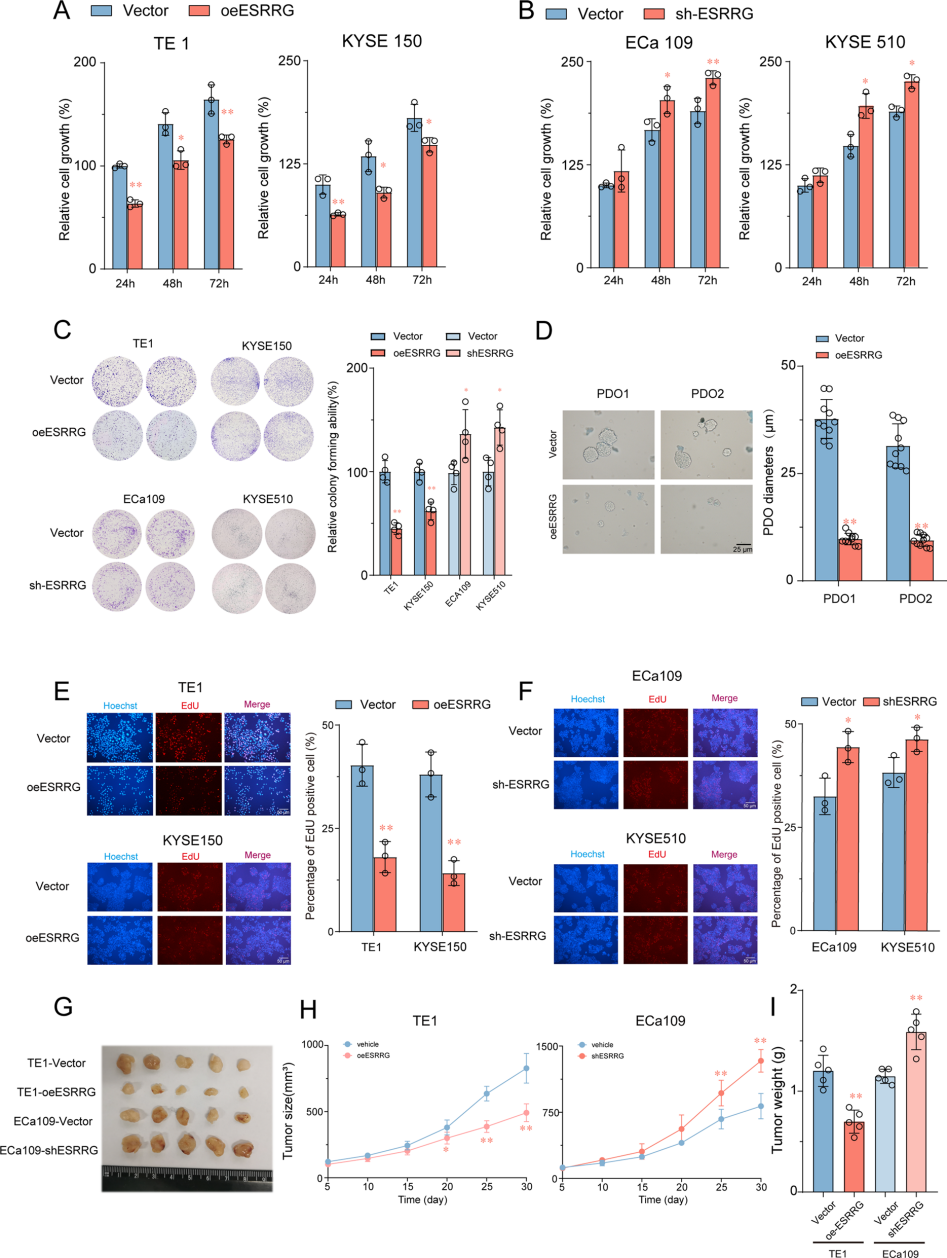
ESRRG suppresses proliferation of ESCC cells in vitro and in vivo. **A**, **B** Cell viability of ESCC cells with ESRRG overexpression or knockdown compared to control vector group were analyzed using was determined by the CCK-8 assay (n = 3). **C** Effects of ESCC cells with ESRRG overexpression and knockdown compared to control vector group on colony formation (n = 4). **D** The representative pictures of two independent ESCC organoids transfected with ESRRG overexpression vectors or control lentivirus for 2 weeks were shown and quantified via organoid diameters (n = 10) **E**, **F** Effects of ESCC cells with ESRRG overexpression and knockdown on EDU assay (n = 3). **G** Image of tumors isolated from nude mice with tumor xenografts derived from the indicated groups. Effects of ESRRG overexpression or knockdown on tumorigenicity in nude mice. **H** Tumour volumes were monitored at indicated time points. **I** The weight of tumours was measured at time of sacrificed (n = 5). Values are presented as mean ± SD. *P < 0.05 or **P < 0.01 indicates significant differences from the vehicle group as assessed by a one-way ANOVA with a post hoc Dunnett’s test

We observed that ESRRG overexpression significantly inhibited cell viability, colony formation, and DNA synthesis in ESCC cells (Fig. 2A, C, E). Conversely, silencing ESRRG significantly promoted the proliferation of ESCC cells compared to the control cells (Fig. 2B, C, F). We also established an ESCC-specific organoid model from two different patients. We found that lentivirus-mediated overexpression of ESRRG markedly inhibited ESCC cell proliferation in the organoids, as quantified by organoid sizes (Fig. 2D).

To assess the tumor suppressor function of ESRRG in vivo, we subcutaneously injected the stable cell lines into nude mice and monitored tumor growth. Consistent with the in vitro results, ESRRG overexpression suppressed tumor growth in nude mice compared to the control group. In contrast, the knockdown of ESRRG significantly enhanced xenograft tumor growth (Fig. 2G). Additionally, tumor volume and weight were remarkably decreased in ESRRG overexpression ESCC cells, while ESRRG knockdown ESCC cells increased tumor volume and weight compared to the control group (Fig. 2H). These in vitro and in vivo findings indicate that ESRRG inhibits the proliferation of ESCC cells and may play a tumor-suppressive role in ESCC.

### ESRRG inhibits ESCC progression by regulating Warburg effect

**Fig. 3:**
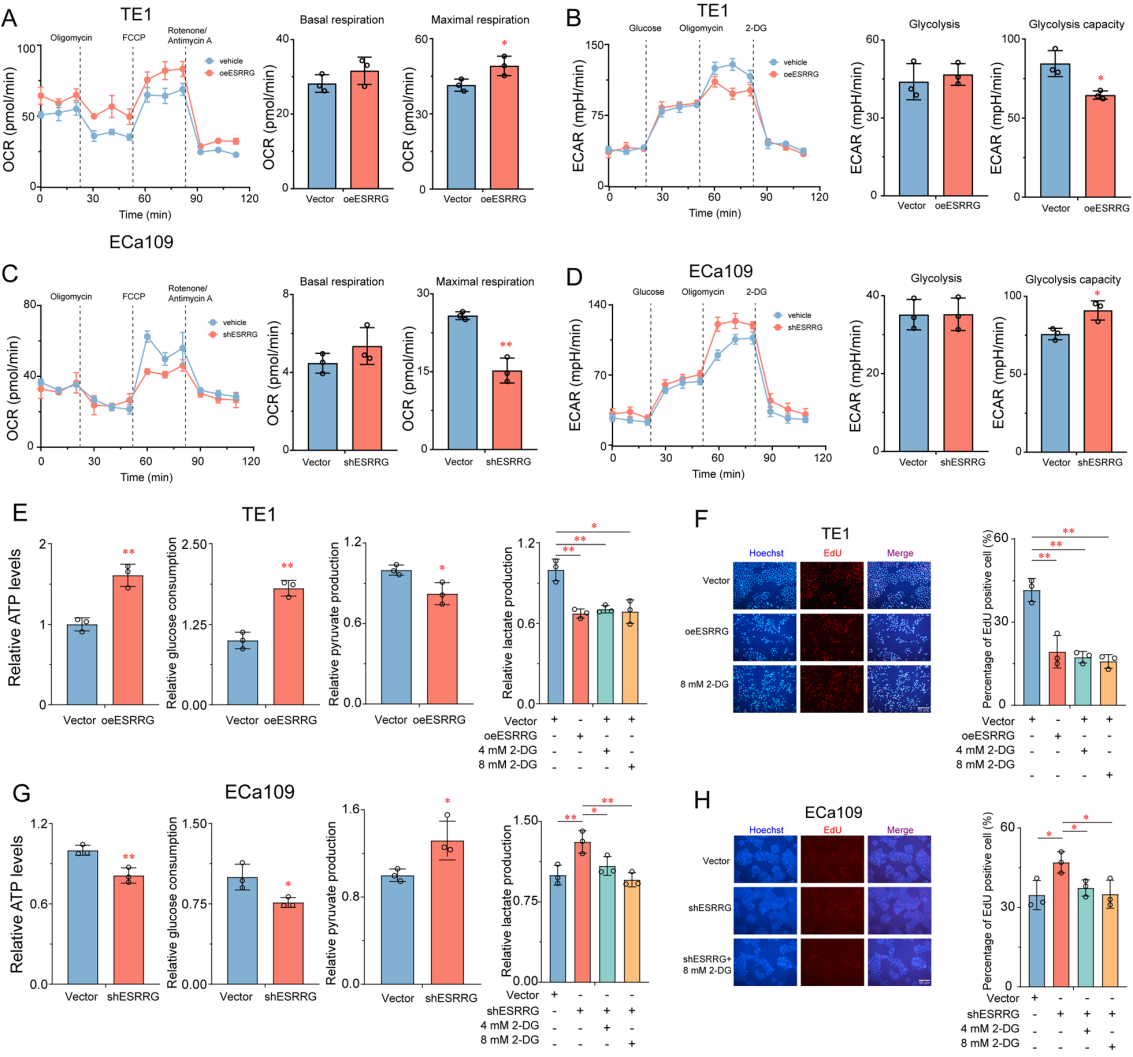
ESRRG inhibits ESCC progression by regulating Warburg effect. **A**–**D** Effects of ESRRG on oxygen consumption ratio (OCR) and extracellular acid ratio (ECAR) in ESCC cells. ATP production, lactate production, pyruvate production and glucose consumption in TE1 cells with ESRRG overexpression **E** and ECa109 cells with ESRRG knockdown **G** compared to relative control cells. **F** Effects of 2-DG on the DNA synthesis of TE1 and ECa109 cells. Values are presented as mean ± SD (n = 3). *P < 0.05 or **P < 0.01 indicates significant differences from the vehicle group as assessed by a one-way ANOVA with a post hoc Dunnett’s test

Previous studies have demonstrated the involvement of ESRRG in the regulation of metabolic reprogramming in various cancer cells [20, 27]. We hypothesized that the inhibition of ESCC cell growth by ESRRG is closely related to its regulation of tumor cell metabolic reprogramming. To test this hypothesis, we compared key cellular metabolic and bioenergetic parameters between ESRRG overexpression ESCC cells or ESRRG knockdown ESCC cells and control cells. The results showed that ESRRG overexpression significantly increased the maximal respiration of TE1 cells by 18.6% and reduced the glycolytic capacity by 23.8% (Fig. 3A, B). Conversely, ESRRG knockout significantly decreased maximal respiration by 40.7% and increased glycolytic capacity by 20.1% in ECa109 cells (Fig. 3C, D). Furthermore, ESRRG overexpression led to increased intracellular ATP levels, glucose uptake, and decreased pyruvate production and extracellular lactate levels in TE1 cells (Fig. 3E). Conversely, ESRRG knockdown decreased intracellular ATP levels, glucose uptake, and increased pyruvate production and extracellular lactate levels (Fig. 3G).

2-DG is widely regarded as a glycolysis inhibitor, making it a valuable tool for investigating the potential involvement of glycolytic effects in the modulation of aberrant cellular functions. To further confirm whether the Warburg effect regulates the growth of ESCC cells, TE1-vector and ECa109-shESRRG cells were treated with different concentrations of 2-DG (0, 4, 8 mM) for 48 h, and the extracellular lactate levels and cell DNA synthesis ability were evaluated. The results demonstrated that 2-DG significantly inhibited the concentration of extracellular lactate levels in both TE1-vector and ECa109-shESRRG cells in a dose-dependent manner (Fig. 3E, G). Moreover, the DNA synthesis of TE1-vector and ECa109-shESRRG cells also decreased with increasing 2-DG concentration (Fig. 3F, H). These findings confirm that ESRRG inhibits tumor cell growth by suppressing the Warburg effect in ESCC cell lines in vitro.

### ESRRG inhibits the expression of key glycolytic enzyme PKM2 in ESCC cells

To gain further insight into the mechanism by which ESRRG regulates glycolytic activity and cell growth in ESCC, we conducted RNA-sequence analysis to examine the transcriptional changes in TE1 cells overexpressing ESRRG. Our data revealed 2829 differentially expressed genes (DEGs), with 1761 genes upregulated and 1068 genes down-regulated in ESRRG overexpression TE1 cells (Log_2_FC > 1 and adjusted p-value < 0.01, Additional file 1: Fig. S4A). We performed gene set enrichment analysis (GSEA) to investigate the impact of these transcriptomic changes on biological pathways and functions. The results indicated a significant negative correlation between ESRRG and the glycolysis pathway in TE1 cells (Additional file 1: Fig. S4B).

**Fig. 4:**
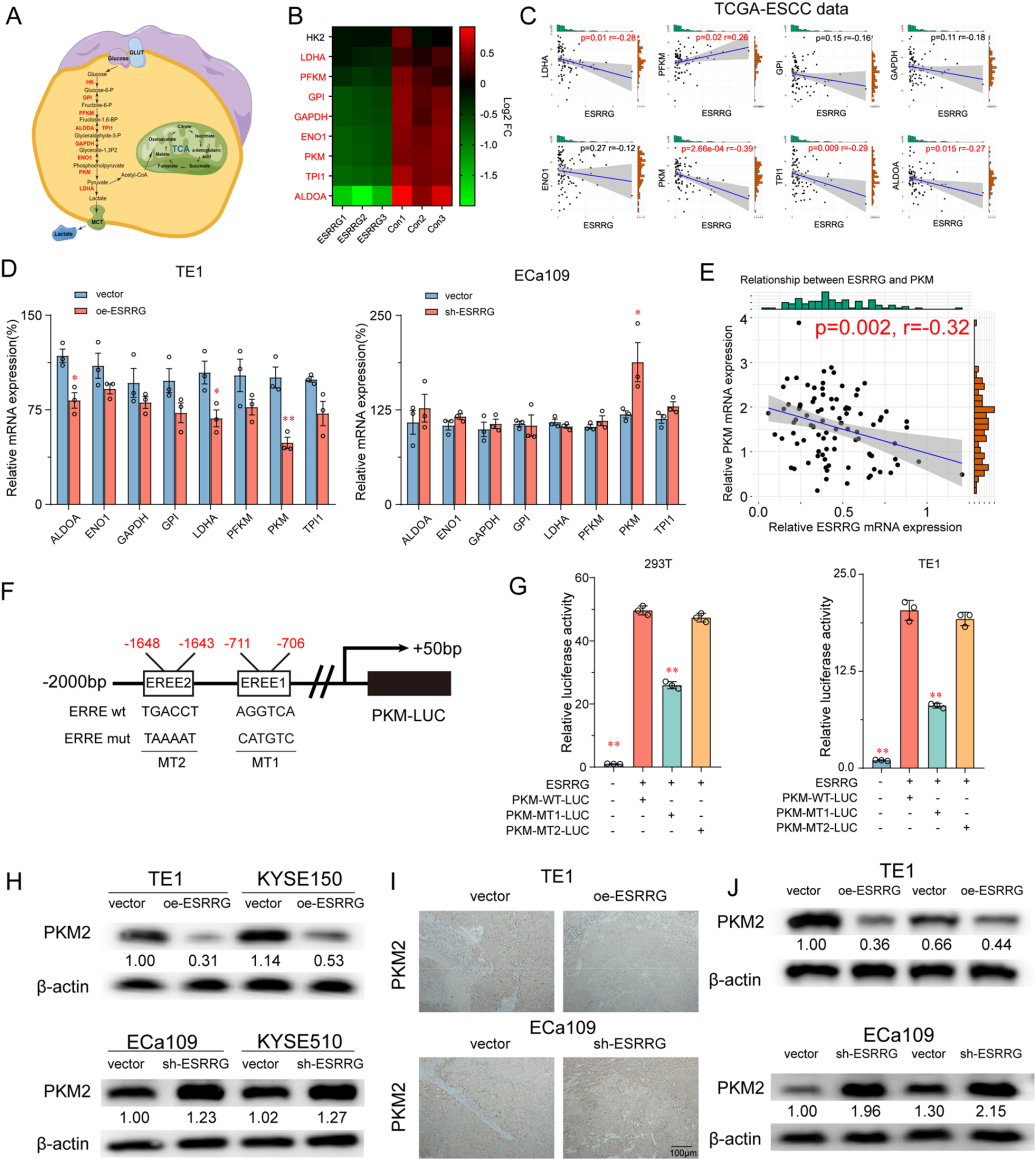
ESRRG inhibits the expression of key glycolytic enzyme PKM2 in ESCC cells. **A** A schematic diagram illustrates the regulation of glycolysis pathway. **B** Heatmap illustrates glycolysis related genes in transcript levels between TE1 cells with ESRRG overexpression and control. **C** The correlation between the relative levels of ESRRG and LDHA, PFKM, GPI, GAPDH, ENO1, PKM, TPI1, ALDOA mRNA transcripts in 82 ESCC tissues of TCGA database. **D** mRNA levels of 8 glycolysis-related genes in TE1 with ESRRG overexpression and ECa109 cells with ESRRG knockdown compared to relative control cells. **E** The correlation between the relative levels of ESRRG and PKM, determined by RT-PCR in 94 ESCC tissues. **F** Schematics of mutation strategies in the PKM promoter (− 2000 bp to + 50 bp). **G** Luciferase reporter assays exhibited that ESRRG bound to the PKM promoter at site 2 to induce its expression. **H** Effects of ESRRG overexpression and knockdown on PKM2 expression in ESCC cells. Immunohistochemistry (**I**) and Western blotting (**J**) for PKM2 protein level in xenograft tumor of mice after overexpression or knockout of ESRRG. Values are presented as mean ± SD (n = 3). *P < 0.05 or **P < 0.01 indicates significant differences from the vehicle group as assessed by a one-way ANOVA with a post hoc Dunnett’s test

Based on these findings, we observed a significant decrease in the expression of eight hub glycolytic genes, including ALDOA, TPI1, PKM, ENO1, GAPDH, GPI, PFKM, and LDHA, upon ESRRG overexpression (Fig. 4A, B). To further explore the relationship between ESRRG and glycolysis-related genes, we analyzed the TCGA database. We found a significant negative correlation between ESRRG mRNA expression and the levels of LDHA, ALDOA, PKM, HK2, TPI1, and GAPDH in ESCC tissues (Fig. 4C).

We specifically focused on the expression of PKM and found that its mRNA levels were prominently altered in TE1 and ECa109 cells following ESRRG overexpression or knockdown (Fig. 4D). Moreover, we investigated the expression of PKM2 in 94 pairs of ESCC tissue specimens. We observed a significant increase in PKM2 mRNA levels in tumor tissues, which exhibited a negative correlation with ESRRG expression (Fig. 4E).

To determine if ESRRG directly induces PKM expression in ESCC cells, we co-transfected the PKM promoter constructs (PKM-WT-Luc, PKM-MT1-Luc, PKM-MT2-Luc) with the ESRRG expression plasmid into 293 T and TE1 cells (Fig. 4F). The luciferase reporter assay revealed that transfection with PKM-WT-Luc significantly enhanced luciferase promoter activity, while transfection with PKM-MT1-Luc, but not PKM-MT2-Luc, significantly reduced luciferase promoter activity (p < 0.01, Fig. 4G). These results strongly indicate that ESRRG directly binds to the PKM promoter and induces PKM transcription in these cell lines.

Furthermore, Western blot analysis showed that ESRRG overexpression significantly decreased the expression of PKM2 in TE1 and KYSE150 cells, whereas silencing ESRRG increased PKM2 expression in KYSE150 and ECa109 cells (Fig. 4H). Immunohistochemical staining and Western blot analysis of xenograft tumor tissues from previous nude mice experiments yielded similar results, with ESRRG overexpression decreasing PKM2 expression and ESRRG knockdown increasing PKM2 expression (Fig. 4I, J). These findings suggest that ESRRG may suppress PKM2 expression to regulate glycolytic activity in ESCC cells.

### PKM is essential for ESRRG to inhibits tumor growth and glycolysis activity in ESCC cells

**Fig. 5:**
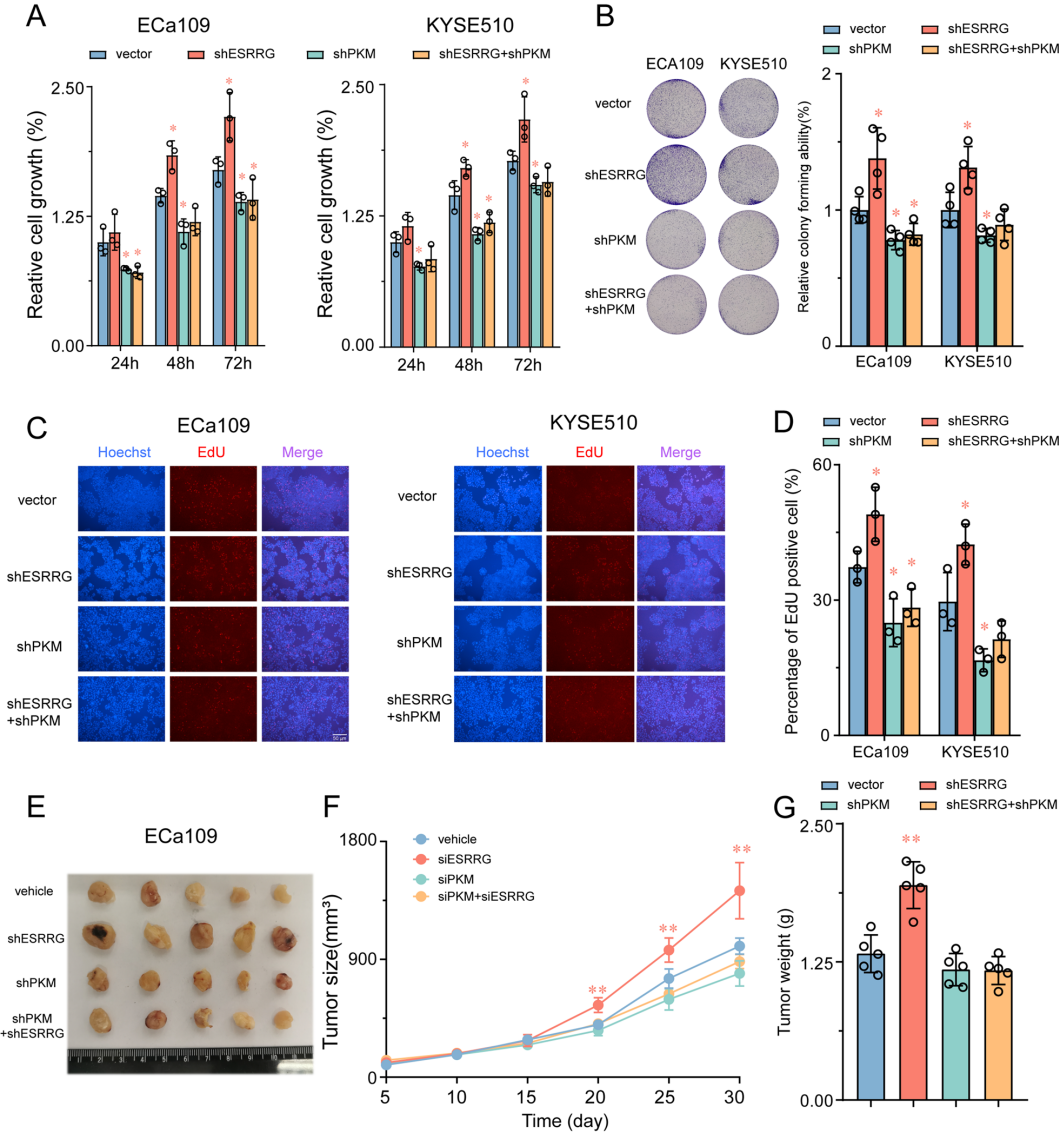
PKM2 is essential for ESRRG to inhibits tumor growth in ESCC cells. **A**–**D** Cell growth were determined in ESRRG knockdown ECa109 and KYSE510 cells with or without further knockdown of PKM employing CCK-8 assays(**A**, colony formation **B** and EDU assay (**C**, **D**). **E** Typical pictures of tumors isolated from nude mice with tumor xenografts derived from the indicated groups. **F** Tumour volumes were monitored at indicated time points. **G** The weight of tumours was measured at time of sacrificed. Values are presented as mean ± SD (n = 3–5). *P < 0.05 or **P < 0.01 indicates significant differences from the vehicle group as assessed by a one-way ANOVA with a post hoc Dunnett’s test

To investigate whether the inhibition of glycolysis activity and tumor growth by ESRRG is mediated through PKM, we performed knockdown of PKM in ECa109 and KYSE510 cells. We confirmed its efficiency by real-time PCR and Western blot (Additional file 1: Fig. S5). As shown in Fig. 5A–C, silencing PKM attenuated the inhibitory effects of ESRRG overexpression on cell viability, colony formation, and DNA synthesis in ESCC cells. Similarly, inhibition of PKM also abrogated the increased glycolytic capacity caused by ESRRG knockdown (Additional file 1: Fig. S6A, B). Moreover, the regulatory effects of ESRRG on intracellular ATP levels, glucose uptake, pyruvate production, and extracellular lactate levels in ESCC cells were significantly weakened after PKM knockdown (Additional file 1: Fig. S6E–H). In an in vivo assay using a xenograft mouse model, the promotion of tumor growth induced by ESRRG knockdown was partially offset by the silencing of PKM (Fig. 5E–G). These results validate that ESRRG suppresses ESCC cell growth by downregulating PKM expression and inhibiting glycolysis activity.

### ESRRG selective agonist, DY131, suppressed glycolysis and tumor growth in ESCC cells

**Fig. 6:**
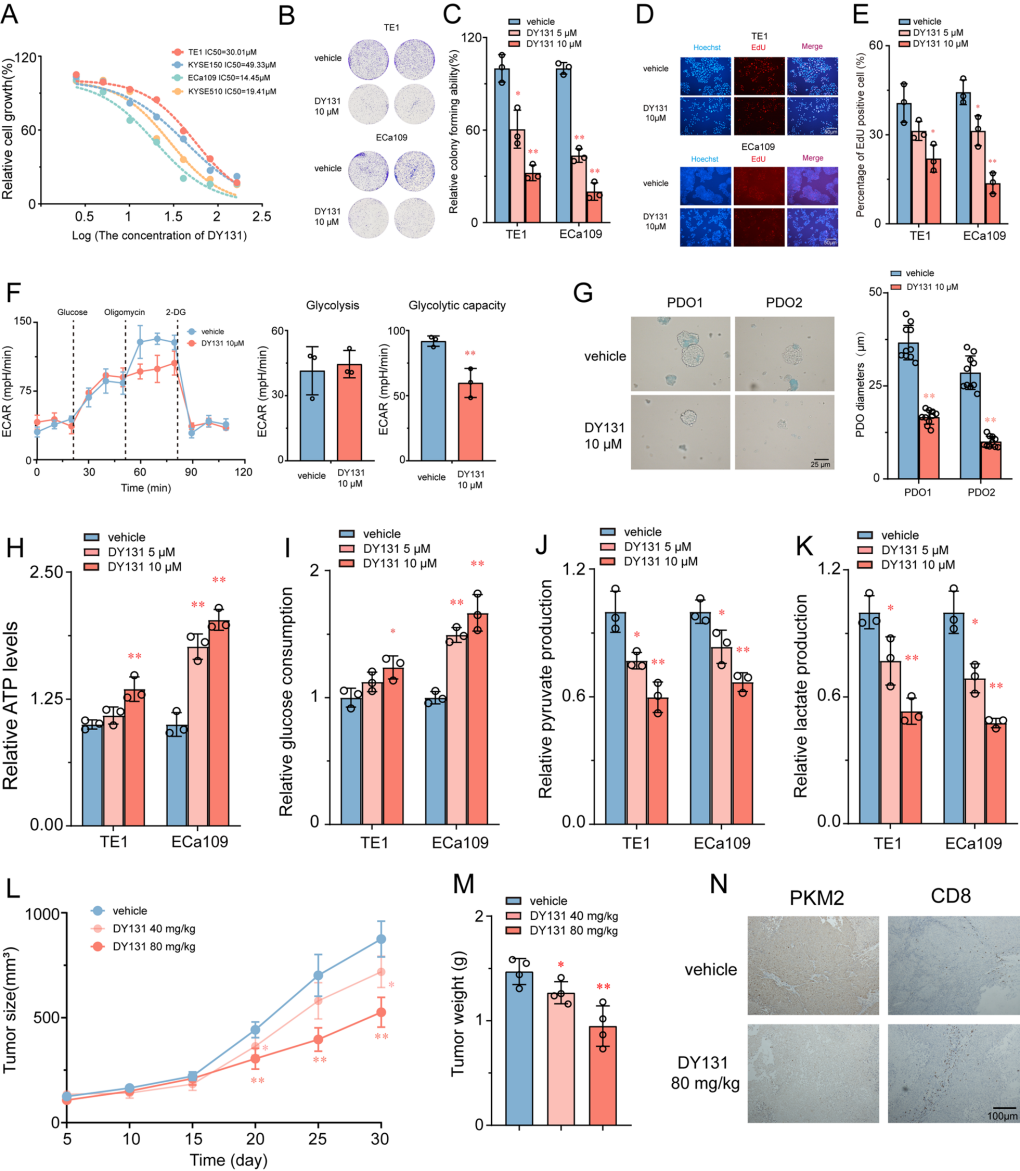
Therapeutic efficacy of the DY131 ESRRG agonist in ESCC. **A** ESCC cells were treated with various concentrations of DY131 for 24 h. Cell viability was determined by the CCK-8 assay. The IC50 value of DY131 in ESCC cell lines was then determined. **B**–**E** Efficacy of DY131 on colony formation (**B**, **C**) and DNA synthesis (**D**, **E**) of ESCC cells (n = 3). **F** Effects of DY131 on extracellular acid ratio (ECAR) in TE1 cell (n = 3). **G** Two different RCC organoids treated with DY131, the diameters kof organoids in two groups were compared. (n = 10). **H**–**K** ECa109 and KYSE510 cells were knocked down for ESRRG and further silenced for PKM, followed by determination of ATP production (**E**), glucose consumption (**F**), pyruvate production (**G**) and lactate production (**H**) (n = 3). **L** After TE1 cell implantation, DY131 or vehicle was intraperitoneally injected into mice every other day and the tumor volume were monitored at indicated time points. **G** The weight of tumours was measured at time of sacrificed (n = 4). **N** Immunohistochemical analysis of mouse samples. Values are presented as mean ± SD. *P < 0.05 or **P < 0.01 indicates significant differences from the vehicle group as assessed by a one-way ANOVA with a post hoc Dunnett’s test

**Fig. 7:**
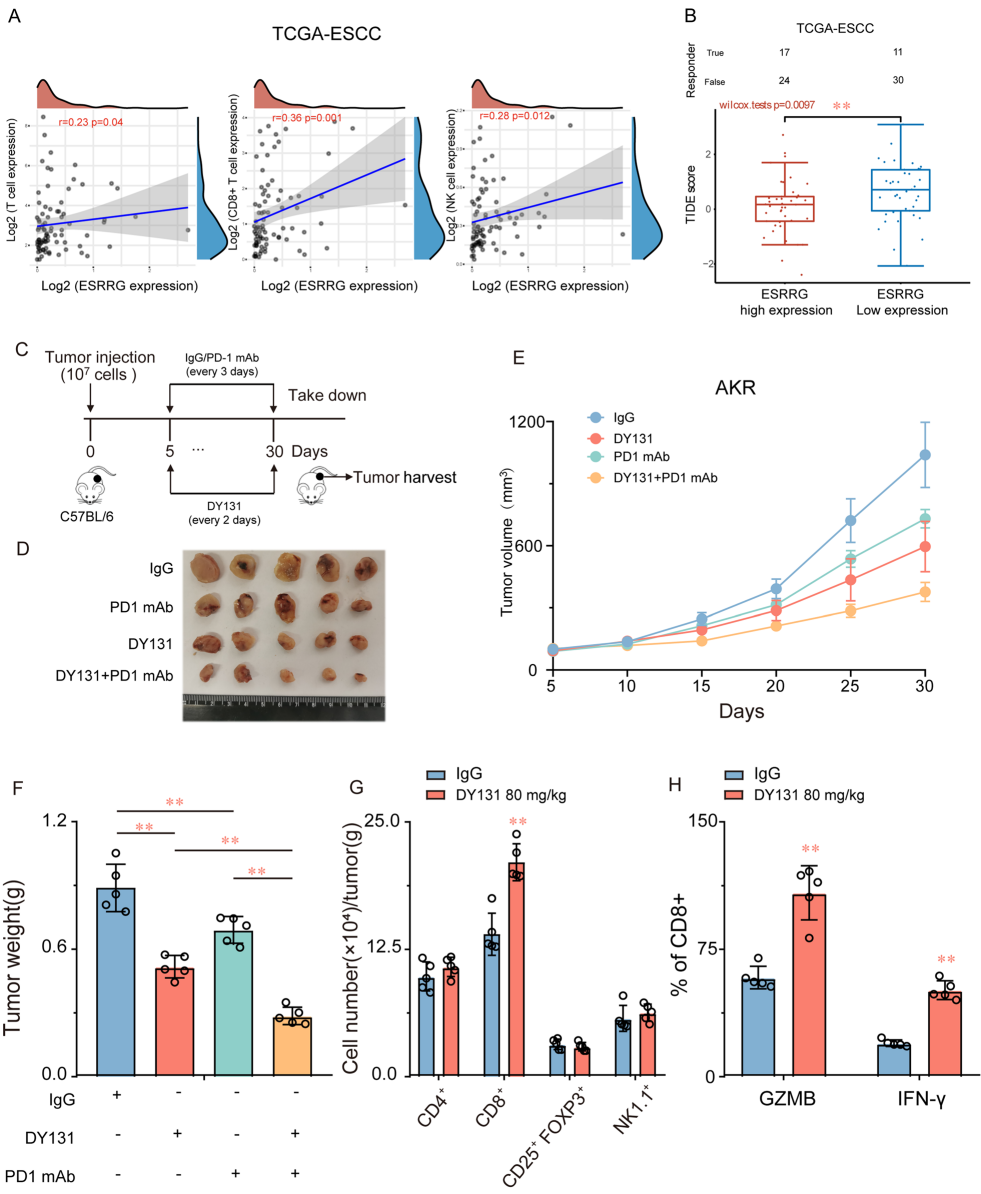
Targeting ESRRG enhances the efficacy of anti–PD-1 therapy. **A** MCPcounter algorithm employed to examine the correlations between ESRRG expression and immune infiltration cells. **B** TIDE scores were lower in the ESRRG high-expression group. **C** Schematic diagram of administration cycles in mice. Tumor growth **D**, **E** and tumor burdens (**F**) of AKR xenografts treated with control (vehicle) or DY131 combined with IgG2a or anti–PD-1 mAb (n = 5). **G**, **H** Quantification of tumor-infiltrating immune cells, GZMB^+^ CD8^+^ cells and IFN-γ^+^ CD8^+^ cells analyzed by flow cytometry using the indicated cell surface markers and in control and DY131 group. *P < 0.05 or **P < 0.01 indicates significant differences from the vehicle group as assessed by a one-way ANOVA with a post hoc Dunnett’s test

Previous results have confirmed that ESRRG effectively inhibits the growth of ESCC cells by inhibiting the glycolysis pathway. Based on this, we evaluated the efficacy of the ESRRG selective agonist DY131 on ESCC cells. The results showed that DY131 exhibited remarkable anti-proliferation activity in ESCC cell lines. We tested the effect of different concentrations of DY131 (0, 6.25, 12.5, 25, 50, 100, 200 μM) on cell viability in ESCC cells for 24 h and measured the IC50 values at 30.01 μM and 14.45 μM for TE1 and ECa109 cells, respectively (Fig. 6A). Similar to the cell viability results, colony formation and EdU assays demonstrated that DY131 (10 μM) significantly suppressed cell colony formation (Fig. 6B, C) and DNA synthesis (Fig. 7D, E) in ESCC cells. The therapeutic effect of DY131 was significantly weakened in ESRRG knockdown cells, confirming that its pharmacological effect was mediated by ESRRG (Additional file 1: Fig. S7D, E). DY131 also remarkably decreased the extracellular acidification rate (ECAR) by 41% and 32% in TE1 cells (Fig. 6D). Additionally, as determined by the sizes of the organoids, DY131 significantly suppressed ESCC organoid growth compared to the control group (Fig. 6G).

In addition, DY131 significantly increased intracellular ATP levels and glucose uptake, and decreased pyruvate production and extracellular lactate levels in ESCC cells (Fig. 6H–K). RT-PCR results confirmed that DY131 suppressed the mRNA expression of glycolysis-associated genes (Additional file 1: Fig. S7A, B). Notably, the tumor suppressor role of DY131 significantly inhibited the expression of PKM2 in ESCC cells, confirming that the ESRRG agonist DY131 inhibited tumor proliferation by acting on the glycolysis pathway (Additional file 1: Fig. S7C). Consistent with the in vitro results, the tumor volume and weight were significantly decreased upon treatment with DY131 in xenograft tumor mice (Fig. 6L, M). Furthermore, immunohistochemical staining of xenograft tumor samples showed that PKM2 expression was significantly reduced, and the number of CD8 + positive cells was significantly increased in the DY131 treatment group (Fig. 6N). In recent years, various glycolysis inhibitors have been proven to have antitumor effects [28]. DY131 exhibits a strong inhibitory effect on ESCC cell proliferation, indicating that glycolytic antagonism via ESRRG can effectively inhibit the growth of ESCC cells. These results confirm that the ESRRG agonist DY131 has potential antitumor activity in ESCC cells by inhibiting the glycolytic enzyme PKM2.

### DY131 enhances the CD8^+^ T cell-mediated antitumor immune response in ESCC

In the previous section, our findings demonstrated a significant increase in the infiltration of CD8-positive cells within the transplanted tumors of tumor-bearing nude mice upon treatment with DY131. These results indicate that DY131 may potentially enhance the antitumor immune response, suggesting that ESRRG could be closely linked to immunotherapy. We assessed the correlation between ESRRG expression in the TCGA-ESCC dataset and immune scores using the MCPcounter software package in R. As illustrated in Fig. 7A, ESRRG was significantly correlated with T cells (R = 0.23, P < 0.05), CD8 + T cells (R = 0.36, P < 0.01), and NK cells (R = 0.28, P < 0.05). Tumor Immune Dysfunction and Exclusion (TIDE) scores indicate sensitivity to immune checkpoint inhibitors; therefore, we evaluated differences in TIDE scores between the ESRRG high expression and low expression groups in the TCGA-ESCC dataset. Our results demonstrated that the TIDE score was significantly lower in the ESRRG high expression group compared with the low expression group (Fig. 7B), indicating that patients with higher ESRRG expression may have better efficacy with immune checkpoint inhibitors.

Based on these findings, we further investigated whether targeting ESRRG could improve the antitumor efficacy of PD-1 blockade. Our results confirmed that DY131 treatment alone at an 80 mg/kg dose reduced tumor growth and weight in AKR tumor-bearing mice (Fig. 7C–F). As anticipated, we also observed that anti-PD-1 monotherapy (100 µg) had a marginal effect on tumor volume and weight. Notably, the combination of DY131 targeting and anti-PD-1 therapy led to further improvements in therapeutic benefits compared with either monotherapy strategy. Next, we collected AKR tumor samples for additional analysis. Flow cytometry results suggested that increased infiltration of CD8 + T cells might be responsible for the enhanced responsiveness observed in the combined treatment groups (Fig. 7G). Moreover, we found that DY131 significantly promoted the secretion of granzyme B (GZMB) and interferon-γ (IFN-γ) by CD8 + T cells (Fig. 7H). Collectively, our findings provide compelling evidence that ESRRG targeting may represent a promising therapeutic strategy to augment the efficacy of anti-PD-1 therapy in ESCC.

### ESRRG negatively correlates with PKM2 in ESCC patients-

**Fig. 8:**
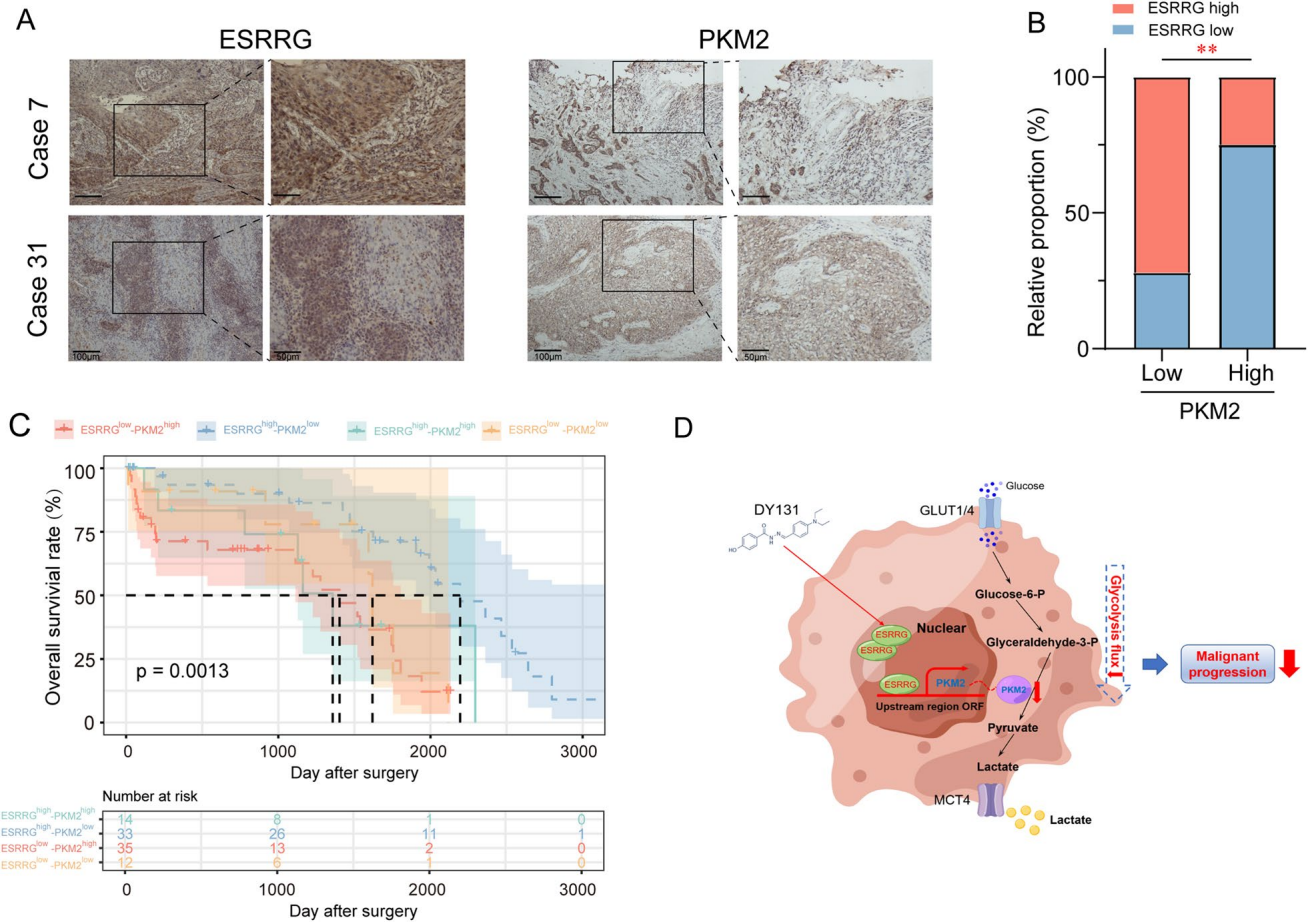
Correlation of ESRRG and PKM2 in ESCC. **A** IHC analysis for ESRRG and PKM2 expression in clinical HCC samples. Scale bars = 100 μm. **B** Correlation between ESRRG expression and PKM2 expression (Chi-squared test). **C** Prognostic value of combining ESRRG and PKM2 levels was analyzed by Kaplan–Meier analysis in 94 ESCC samples. **D** Model illustrating tumor suppression mechanism regulated by ESRRG in ESCC progression

To explore the prognostic values of the ESRRG-PKM2 axis in ESCC patients, an IHC analysis was performed on 94 patients who underwent surgical resection to determine the expression levels of ESRRG and PKM2. Representative photographs of ESRRG and PKM2 in ESCC tissues are shown in Fig. 8A. PKM2 staining was weak in ESCC tissues with high ESRRG expression, while it was strong in tissues with low ESRRG expression. We analyzed the associations between ESRRG expression and PKM2 expression in the 94 ESCC samples. The results showed a significant negative correlation between ESRRG expression and PKM2 expression (Fig. 8B). the 94 ESCC samples were also analyzed for PKM2 and ESRRG expression levels to determine their prognostic value. Kaplan–Meier analysis demonstrated that ESCC patients with high ESRRG expression and low PKM2 expression had the highest overall survival (Fig. 8C). These findings suggest that PKM2 plays a role in ESRRG-mediated ESCC progression.

## Discussion

Previous studies have established a close association between the ERR family of transcription factors and cancer development and progression [29]. For example, ESRRG has been shown to antagonize the proliferation of gastric cancer cells by suppressing TCF4/LEF1 binding to the CCND1 promoter, highlighting its tumor suppressor role [21, 30]. In our study, we investigated the tumor suppressor role of ESRRG in ESCC and its regulation of the Warburg effect through the ESRRG-PKM2 axis.

Metabolic reprogramming, known as the Warburg effect, plays a crucial role in cancer development, providing tumors with the energy and resources needed for proliferation and metastasis [31, 32]. Previous studies have demonstrated the involvement of glycolytic enzymes and regulators, such as ras-related C3 botulinum toxin substrate 1 and PKM2, in promoting ESCC cell proliferation [33, 34]. In our study, we observed that overexpression of ESRRG suppressed the proliferation and glycolytic activity of ESCC cells, while ESRRG silencing had the opposite effect. These findings are consistent with previous studies highlighting the role of ESRRG in metabolic reprogramming in other cancer types [20]. Thus, targeting glycolysis activity through ESRRG inhibition may hold therapeutic potential for ESCC patients.

PKM2, an enzyme involved in the final step of glycolysis, is highly expressed in various cancers and is associated with poor prognosis [33–35]. Our data confirm the overexpression of PKM2 in ESCC cells and tissues, which correlates with poor overall survival. Interestingly, we found a strong negative correlation between PKM2 and ESRRG expression in ESCC, further validated using public databases. We also demonstrated that ESRRG can directly bind to the PKM promoter region and interfere with its activity, leading to the downregulation of PKM2 expression. Furthermore, ESRRG-mediated suppression of PKM2 expression resulted in reduced glycolysis activity and inhibited the survival, proliferation, and DNA synthesis of ESCC cells. Overall, our findings suggest that the ESRRG-PKM2 axis inhibits ESCC progression by regulating tumor cell metabolic reprogramming.

Our present results indicate that ESRRG functions as a negative transcriptional regulator of the glycolysis pathway, and overexpression of ESRRG in ESCC cells significantly inhibits the transcriptional translation of several key enzymes in the glycolysis pathway, such as hexokinase 2 (HK2), aldolase A (ALDOA), enolase 1 (ENO1), pyruvate kinase M2 (PKM2), and lactate dehydrogenase A (LDHA). As such, we hypothesize that ESRRG's active agonist, DY131, may effectively inhibit the glycolytic pathway and provide a potential therapeutic approach for ESCC. In recent years, with advances in research on nuclear receptors’ function, their roles in modulating gene transcription are considered potential targets for tumor therapy. Therefore, nuclear receptor agonists or antagonists are widely used in cancer treatment. For example, anti-estrogen tamoxifen is a first-line breast cancer drug for both pre- and post-menopausal patients [36]. Additionally, apalutamide, a second-generation androgen receptor antagonist, is used for the treatment of non-metastatic castration-resistant prostate cancer [37]. The ESRRG-specific agonist DY131 has been found to suppress gastric carcinoma and prostate cancer cell proliferation [20, 21]. These results suggest that ESRRG is a key factor regulating the glycolytic pathway, and the development of more selective ESRRG agonists will provide novel approaches to improve ESCC outcomes.

Lactate production by tumor cells is known to adversely affect immune cell function, reducing the efficacy of PD-1 inhibitors [38, 39]. Additionally, excessive lactate levels can promote tumor cell proliferation, invasion, and metastasis, impeding the infiltration and activity of immune cells [40]. Thus, the effect of lactate levels on the efficacy of PD-1 inhibitors has been a topic of great interest. Currently, researchers discuss various strategies proposed to overcome the impact of lactate levels on PD-1 inhibitor therapy. Studies suggest that the combined use of LDHA and PD-1 inhibitors could improve the latter's efficacy by reducing the amount of lactate produced by tumor cells [41]. Another strategy is using lactate transferase 4 (MCT4) inhibitors to counteract the impact of lactate in the tumor microenvironment [42]. However, the efficacy of these combined therapies requires further research, and their long-term impacts are yet to be determined. In this study, we have observed that the overexpression of ESRRG or the administration of DY131 has the potential to significantly inhibit lactate production in tumor cells. Additionally, this treatment strategy effectively enhanced the antitumor effect of PD-1 inhibitors. These results have provided novel evidence to support the efficacy of combination therapy for enhancing the clinical therapeutic effect of PD-1 inhibitors. Our findings hold significant implications for future investigation and the development of therapeutic interventions in cancer treatment.

## Conclusion

Our study has uncovered the significant roles and underlying mechanisms of ESRRG in regulating the proliferation and metabolism of esophageal squamous cell carcinoma (ESCC). Our findings demonstrate that ESRRG plays a crucial role in inhibiting the proliferation of ESCC cells both in vitro and in vivo by suppressing aerobic glycolysis, primarily through its interaction with PKM2, a key enzyme in the glycolytic pathway. Furthermore, we have provided compelling evidence supporting the direct regulation of PKM transcription by ESRRG through its binding to the promoter region of the PKM gene. These groundbreaking discoveries provide novel insights into the molecular mechanisms through which ESRRG exerts its inhibitory effects on ESCC development, opening up possibilities for developing targeted pharmacotherapies aimed at ESRRG and its downstream targets.

### Supplementary Information

**Additional file 1:** **Figure S1.** ESRRA and ESRRB expression in paired samples of tumor tissues and nontumorous adjacent normal esophagus tissues from patients with esophageal squamous cell carcinoma (ESCC) in the TCGA and three GSE cohorts. **Figure S2.** Expression levels of ESRRG in ESCC cell lines. The expression levels of ESRRG were measured by real-time PCR analysis (**A**) and western blot analysis (**B**) in a panel of ESCC cell lines. All data are shown as the mean±SD of 3 independent experiments. *P< 0.05, **P< 0.01. **Figure S3.** Expression levels of ESRRG in ESCC cells stably infected with recombinant lentivirus. mRNA levels (**A**, **B**) of ESRRG were detected by real-time PCR in ESCC cells infected with ESRRG overexpression or control lentivirus (**A**), ESRRG knockdown or control lentivirus (**B**). Protein levels (**C**, **D**) of ESRRG were detected by western blot to detect the overexpression or knockdown efficiency in ESCC cells. (**E**, **F**) Overexpression or knockdown ESRRG efficiency in ESCC cells was verified by observing the intensity of green fluorescence after cell transfection with a tool virus-bearing fluorescent reporter gene. Statistical tests: unpaired two-tailed Student’s t-test (**A**, **B**). All data are shown as the mean ± SD of 3 independent experiments. **P< 0.05. **Figure S4. A** Volcano plot illustrating the global difference between TE1 cells with ESRRG overexpression and control cells. **B** Gene set enrichment analysis (GSEA) indicate a significant change of glycolysis signaling induced by ESRRG. NES, normalized enrichment score. **Figure S5.** Expression levels of PKM in ESCC cells stably infected with recombinant lentivirus. mRNA levels (**A**) of PKM were detected by real-time PCR in ESCC cells infected with PKM knockdown or control lentivirus. Protein levels (**B**) of PKM2 were detected by western blot to detect the knockdown efficiency in ESCC cells. Statistical tests: unpaired two-tailed Student’s t-test (**A**, **B**). All data are shown as the mean ± SD of 3 independent experiments. **P< 0.05. **Figure S6.** The effects of ESRRG on aerobic glycolysis in ESCC cells are dependent on PKM. (**A**, **B**) ECa109 cells were detected for ECAR to indicate glycolysis flux and glycolytic capacity. (**C**, **D**) The OCR was detected to indicate basal respiration and maximal respiration. (**E**–**H**) ECa109 and KYSE510 cells were knocked down for ESRRG and further silenced for PKM, followed by determination of ATP production (E), glucose consumption (F), pyruvate production (G) and lactate production (H). Values are presented as mean ± SD (n=3). *P < 0.05 or **P < 0.01 indicates significant differences from the vehicle group as assessed by a one-way ANOVA with a post hoc Dunnett’s test. **Figure S7. **(**A**, **B**) TE1 and ECa109 cell treated with or without 10 μM DY131 for 48 hours. The expression of glycolysis-related gene was examined by RT-PCR. (C) Western blot analysis for PKM2 after treatment of DY131 in ESCC cells. The therapeutic efficacy of the DY131were determined in TE1 and ECa109 cells with or without further knockdown of ESRRG employing CCK-8 assays (D) and colony formation (**E**). All data are shown as the mean ± SD of 3 independent experiments. **P< 0.05. **Figure S8. **No major obvious organ toxicity observed in the vital organs of the DY131 treated mice (A) H&E staining sections of organs including heart, liver, spleen, lung and kidney from the different groups (**B**) Weight of final dissected organs. Data are mean ± SD (n=5). **Figure S9. **Gating strategy for flow cytometry analysis of lymphoid and myeloid population in AKR tumors. **Table S1.** Primer sequences of genes in qRT -PCR assay.

## Data Availability

The datasets generated and/or analyzed during the current study are included in this published article (and its supplementary information files) and all the raw data available from the corresponding author on reasonable request.

## Abbreviations

ALDOA: Aldolase A
CCND1: Cyclin D1
ESRRG: Estrogen-related receptor gamma
ECAR: Extracellular acidification rate
ENO1: Enolase 1
ESCC: Esophageal squamous cell carcinoma
GAPDH: Glyceraldehyde-3-phosphate dehydrogenase
GPI: Glucose-6-phosphate isomerase
GRIP1: Glucocorticoid receptor interacting protein 1
HK2: Hexokinase 2
IHC: Immunohistochemistry
LDHA: Lactate dehydrogenase A
OCR: Oxygen consumption rate
PD-1: Programmed Death 1
PFKM: Phosphofructokinase
PKM2: Pyruvate kinase M2
RT-PCR: Real-time reverse transcription polymerase chain reaction
TCGA: The Cancer Genome Atlas Program
TPI1: Triosephosphate Isomerase 1
