# Supplementary material for: ESRRG-PKM2 axis reprograms metabolism to suppress esophageal squamous carcinoma progression and enhance anti-PD-1 therapy efficacy
Source: J Transl Med. 2023 Sep 7;21:605. doi: 10.1186/s12967-023-04347-5 (PMC10485992; doi:10.1186/s12967-023-04347-5)
Supplement: Supplementary file 1 — Additional file 1: Figure S1. ESRRA and ESRRB expression in paired samples of tumor tissues and nontumorous adjacent normal esophagus tissues from patients with esophageal squamous cell carcinoma (ESCC) in the TCGA and three GSE cohorts. Figure S2. Expression levels of ESRRG in ESCC cell lines. The expression levels of ESRRG were measured by real-time PCR analysis (A) and western blot analysis (B) in a panel of ESCC cell lines. All data are shown as the mean±SD of 3 independent experiments. *P< 0.05, **P< 0.01. Figure S3. Expression levels of ESRRG in ESCC cells stably infected with recombinant lentivirus. mRNA levels (A, B) of ESRRG were detected by real-time PCR in ESCC cells infected with ESRRG overexpression or control lentivirus (A), ESRRG knockdown or control lentivirus (B). Protein levels (C, D) of ESRRG were detected by western blot to detect the overexpression or knockdown efficiency in ESCC cells. (E, F) Overexpression or knockdown ESRRG efficiency in ESCC cells was verified by observing the intensity of green fluorescence after cell transfection with a tool virus-bearing fluorescent reporter gene. Statistical tests: unpaired two-tailed Student’s t-test (A, B). All data are shown as the mean ± SD of 3 independent experiments. **P< 0.05. Figure S4. A Volcano plot illustrating the global difference between TE1 cells with ESRRG overexpression and control cells. B Gene set enrichment analysis (GSEA) indicate a significant change of glycolysis signaling induced by ESRRG. NES, normalized enrichment score. Figure S5. Expression levels of PKM in ESCC cells stably infected with recombinant lentivirus. mRNA levels (A) of PKM were detected by real-time PCR in ESCC cells infected with PKM knockdown or control lentivirus. Protein levels (B) of PKM2 were detected by western blot to detect the knockdown efficiency in ESCC cells. Statistical tests: unpaired two-tailed Student’s t-test (A, B). All data are shown as the mean ± SD of 3 independent experiments. **P< 0.05. Fig [file 12967_2023_4347_MOESM1_ESM.docx]

**Additional file results**


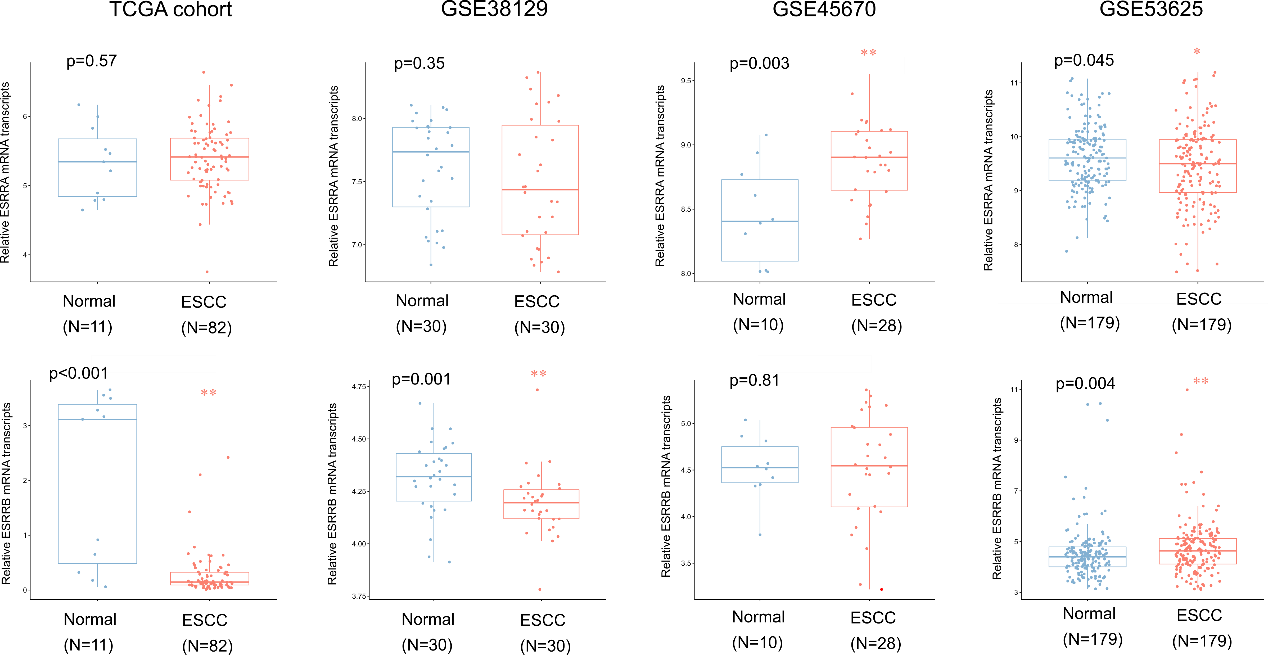


**Figure S1** ESRRA and ESRRB expression in paired samples of tumor tissues and nontumorous adjacent normal esophagus tissues from patients with esophageal squamous cell carcinoma (ESCC) in the TCGA and three GSE cohorts.


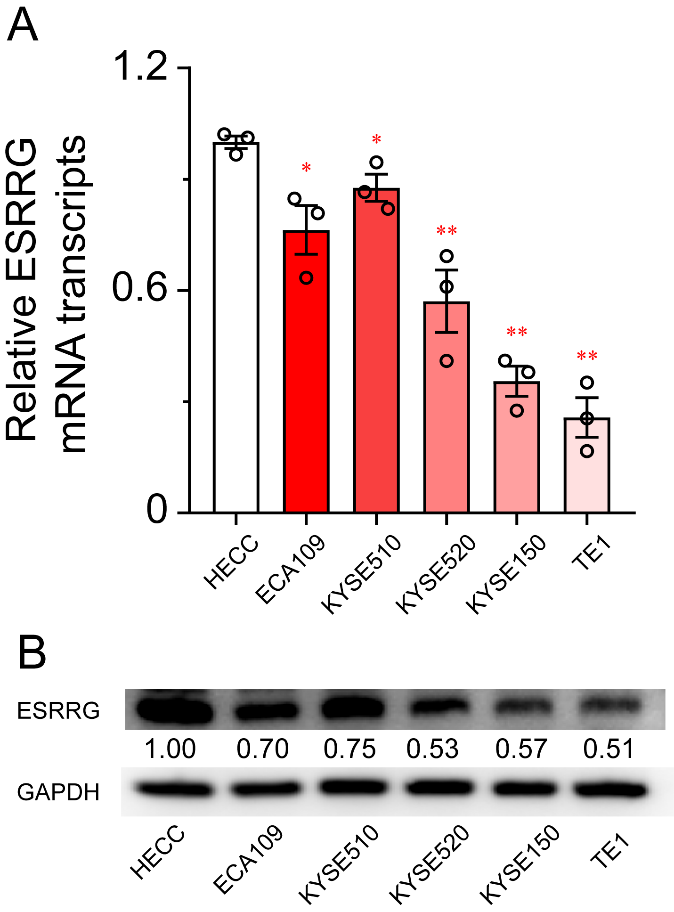


**Figure S2** Expression levels of ESRRG in ESCC cell lines. The expression levels of ESRRG were measured by real-time PCR analysis (A) and western blot analysis (B) in a panel of ESCC cell lines. All data are shown as the mean±SD of 3 independent experiments. *P< 0.05, **P< 0.01


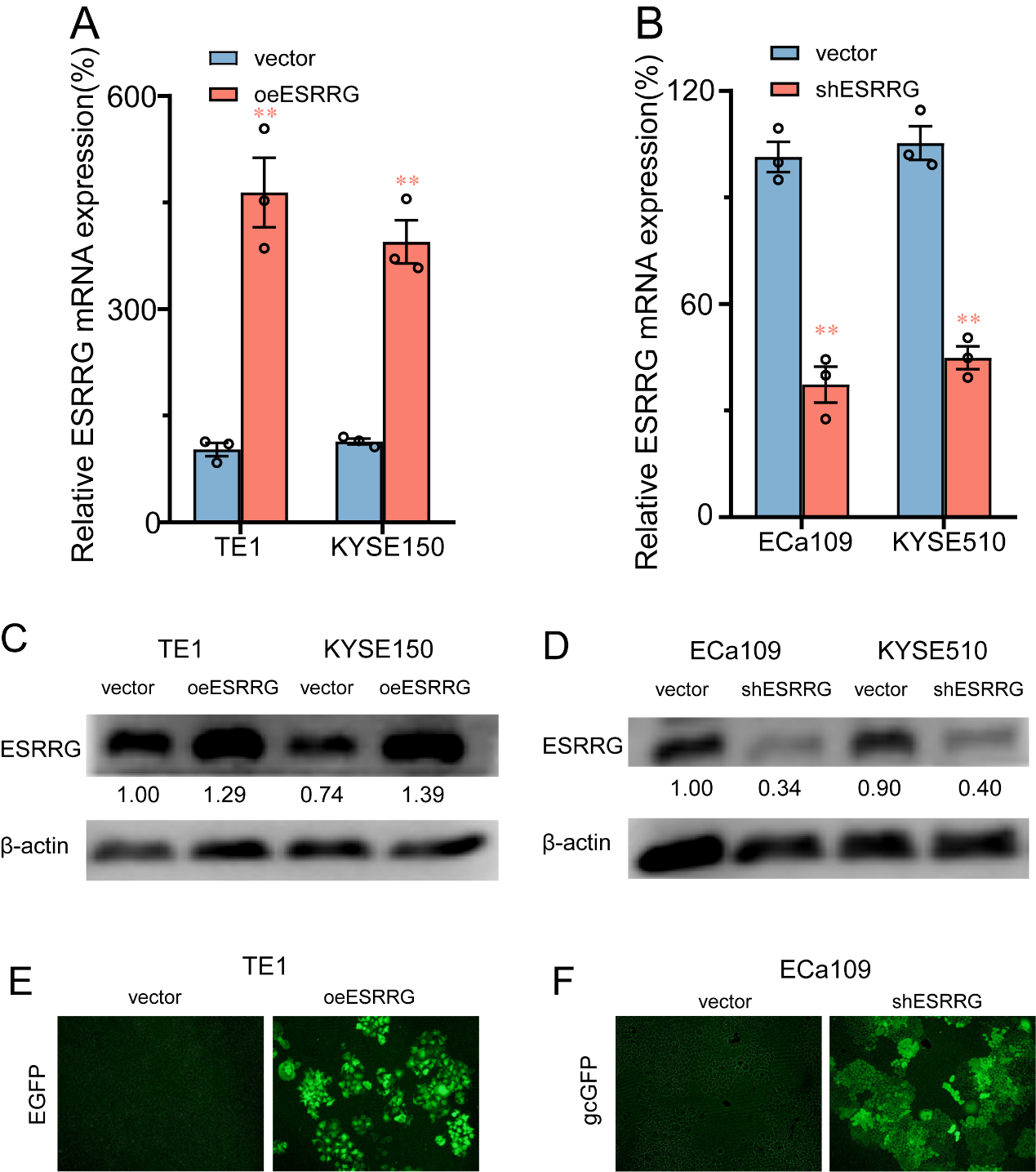


**Figure S3**. Expression levels of ESRRG in ESCC cells stably infected with recombinant lentivirus. mRNA levels (A, B) of ESRRG were detected by real-time PCR in ESCC cells infected with ESRRG overexpression or control lentivirus (A), ESRRG knockdown or control lentivirus (B). Protein levels (C, D) of ESRRG were detected by western blot to detect the overexpression or knockdown efficiency in ESCC cells. (E, F) Overexpression or knockdown ESRRG efficiency in ESCC cells was verified by observing the intensity of green fluorescence after cell transfection with a tool virus-bearing fluorescent reporter gene. Statistical tests: unpaired two-tailed Student’s t-test (A-B). All data are shown as the mean ± SD of 3 independent experiments. **P< 0.05.


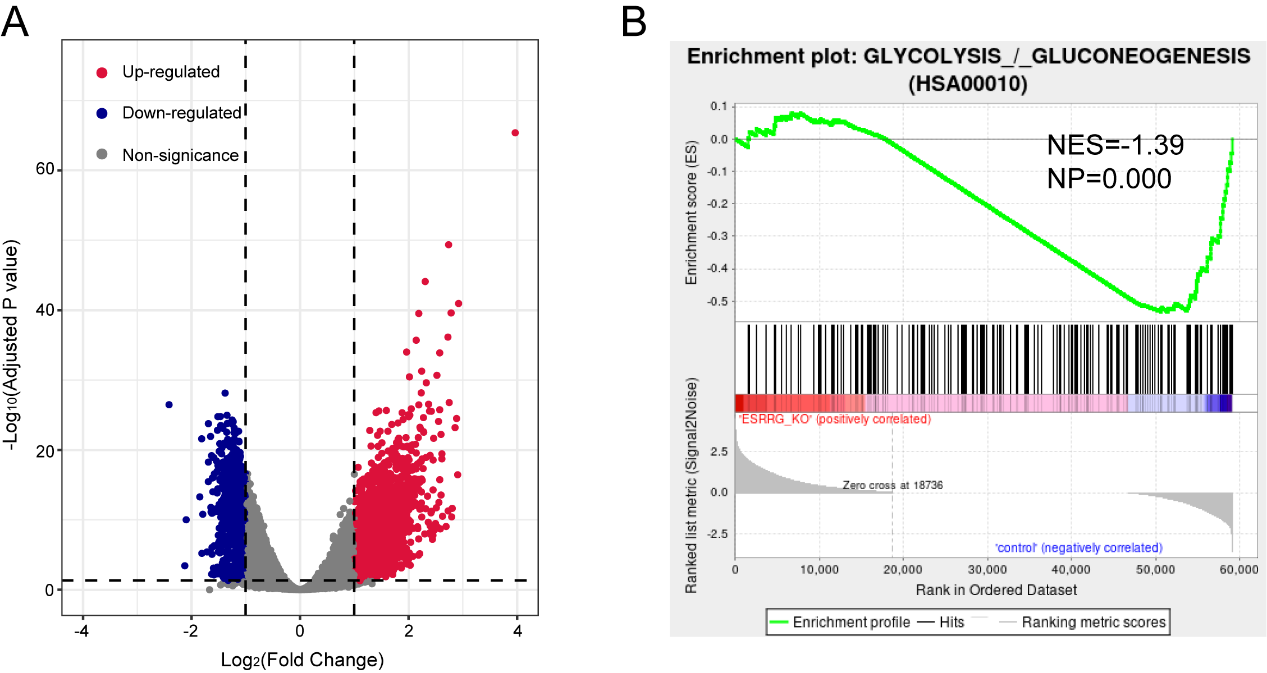


**Figure S4** (A)Volcano plot illustrating the global difference between TE1 cells with ESRRG overexpression and control cells. (B) Gene set enrichment analysis (GSEA) indicate a significant change of glycolysis signaling induced by ESRRG. NES, normalized enrichment score.


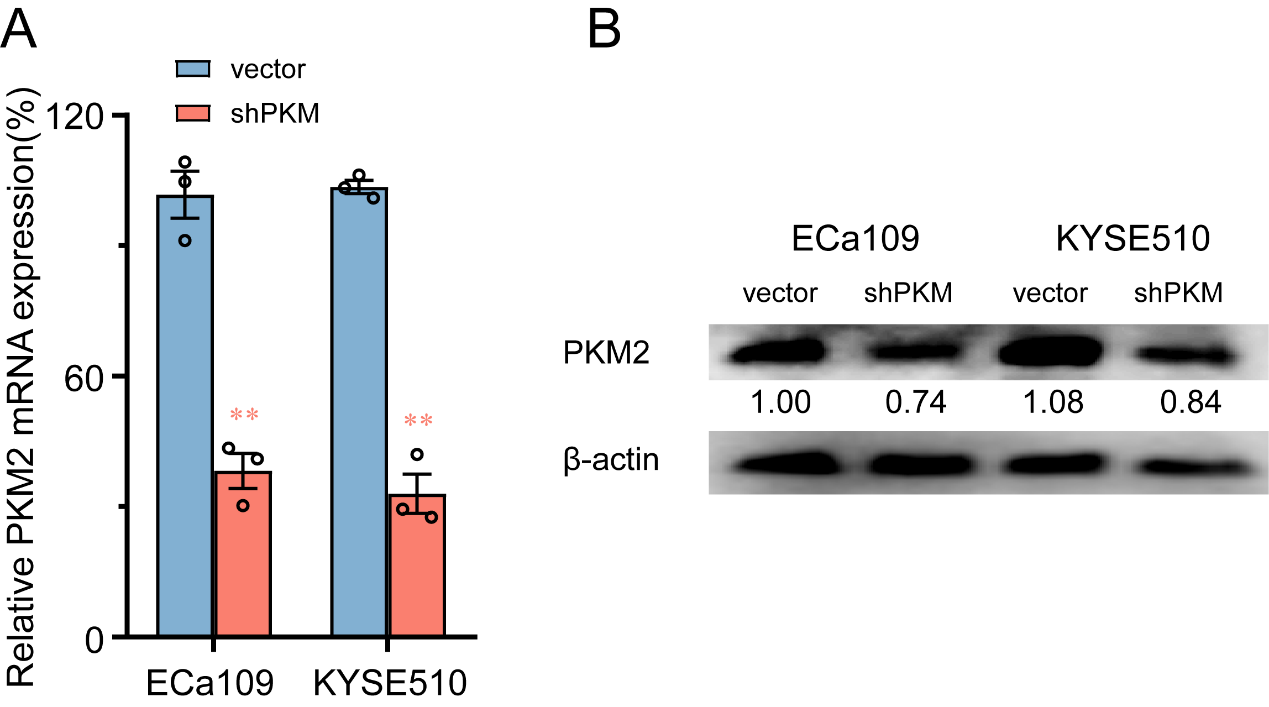


**Figure S5**. Expression levels of PKM in ESCC cells stably infected with recombinant lentivirus. mRNA levels (A) of PKM were detected by real-time PCR in ESCC cells infected with PKM knockdown or control lentivirus. Protein levels (B) of PKM2 were detected by western blot to detect the knockdown efficiency in ESCC cells. Statistical tests: unpaired two-tailed Student’s t-test (A-B). All data are shown as the mean ± SD of 3 independent experiments. **P< 0.05.


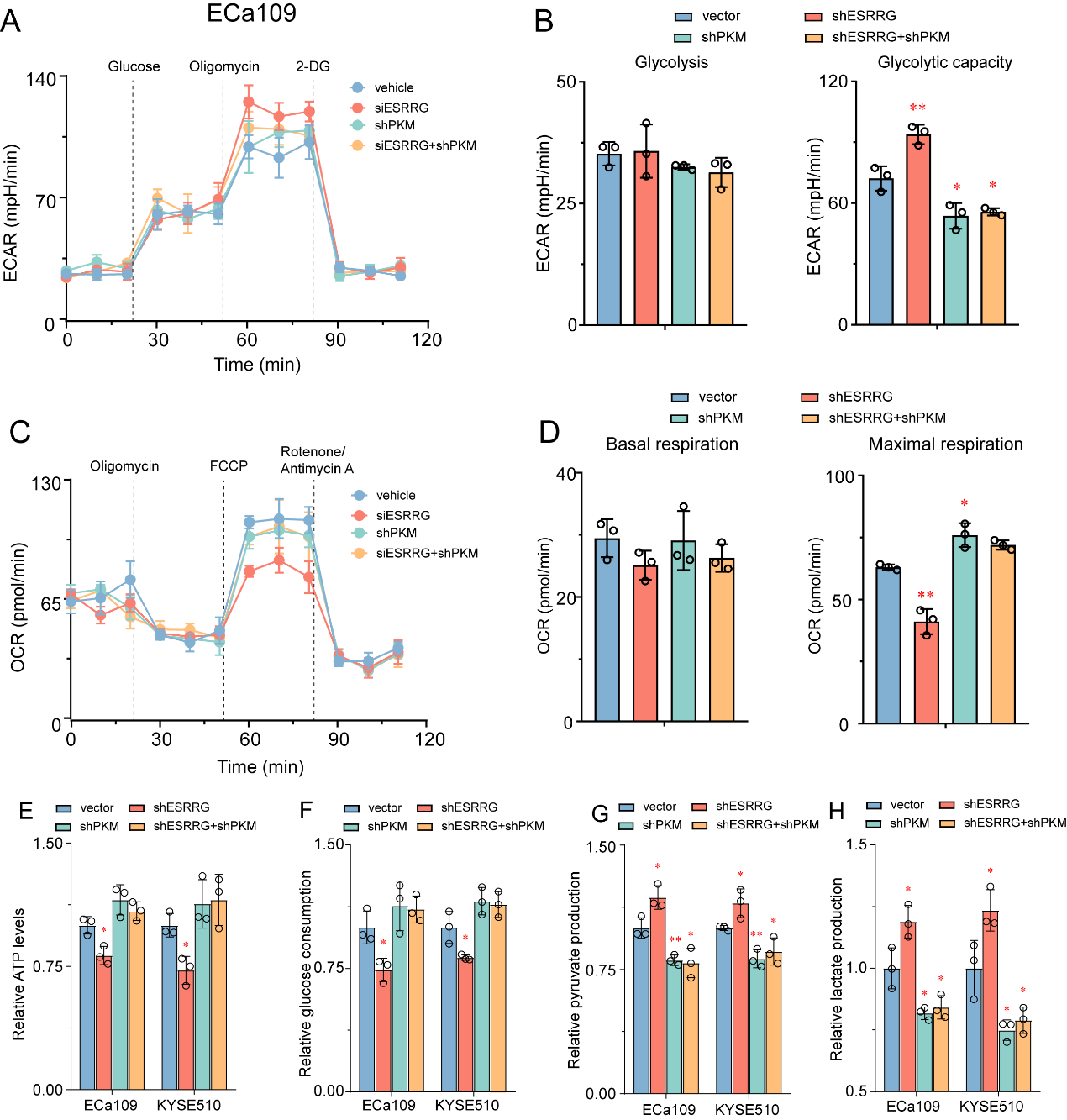


**Figure S6 The effects of ESRRG on aerobic glycolysis in ESCC cells are dependent on PKM.** (A, B) ECa109 cells were detected for ECAR to indicate glycolysis flux and glycolytic capacity. (C, D) The OCR was detected to indicate basal respiration and maximal respiration. (E-H) ECa109 and KYSE510 cells were knocked down for ESRRG and further silenced for PKM, followed by determination of ATP production (E), glucose consumption (F), pyruvate production (G) and lactate production (H). Values are presented as mean ± SD (n=3). *P < 0.05 or **P < 0.01 indicates significant differences from the vehicle group as assessed by a one-way ANOVA with a post hoc Dunnett’s test.


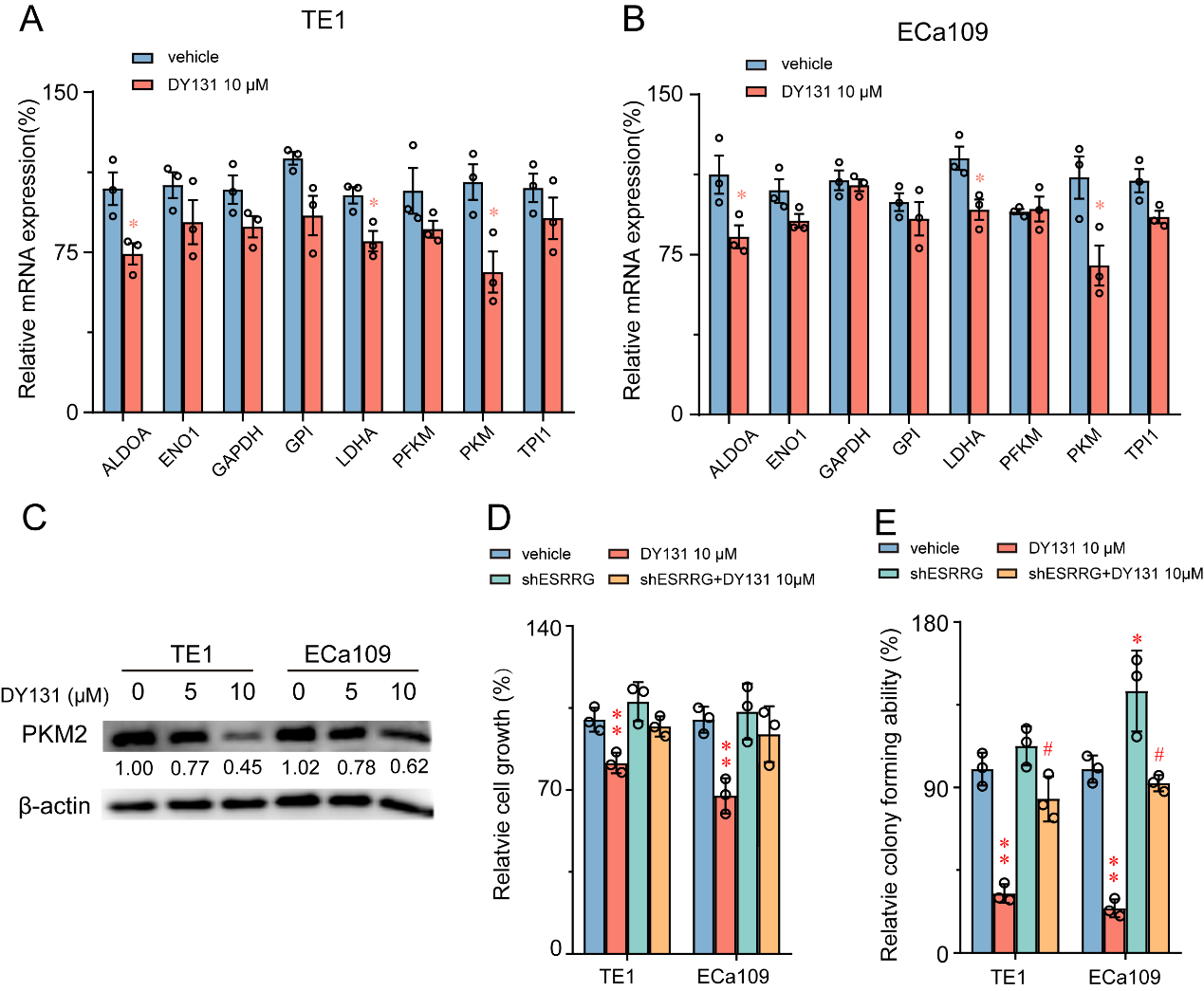


**Figure S7** (A, B) TE1 and ECa109 cell treated with or without 10 μM DY131 for 48 hours. The expression of glycolysis-related gene was examined by RT-PCR. (C) Western blot analysis for PKM2 after treatment of DY131 in ESCC cells. The therapeutic efficacy of the DY131were determined in TE1 and ECa109 cells with or without further knockdown of ESRRG employing CCK-8 assays (D) and colony formation (E). All data are shown as the mean ± SD of 3 independent experiments. **P< 0.05.


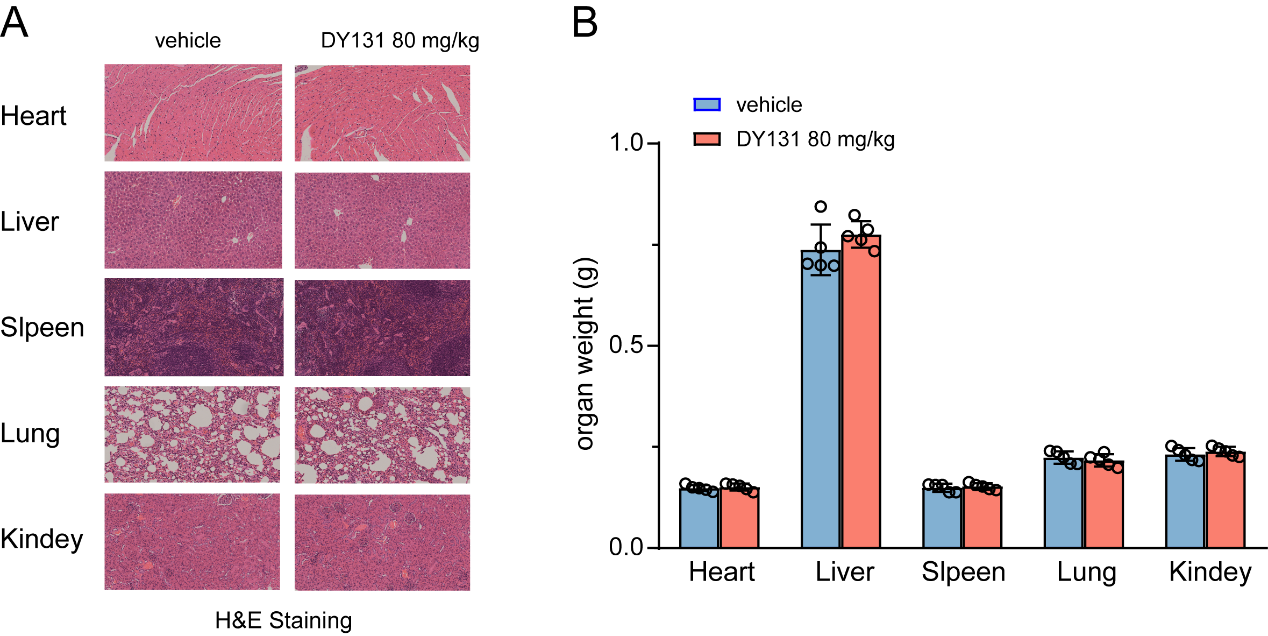


**Figure S8** No major obvious organ toxicity observed in the vital organs of the DY131 treated mice (A) H&E staining sections of organs including heart, liver, spleen, lung and kidney from the different groups (B) Weight of final dissected organs. Data are mean ± SD (n=5).


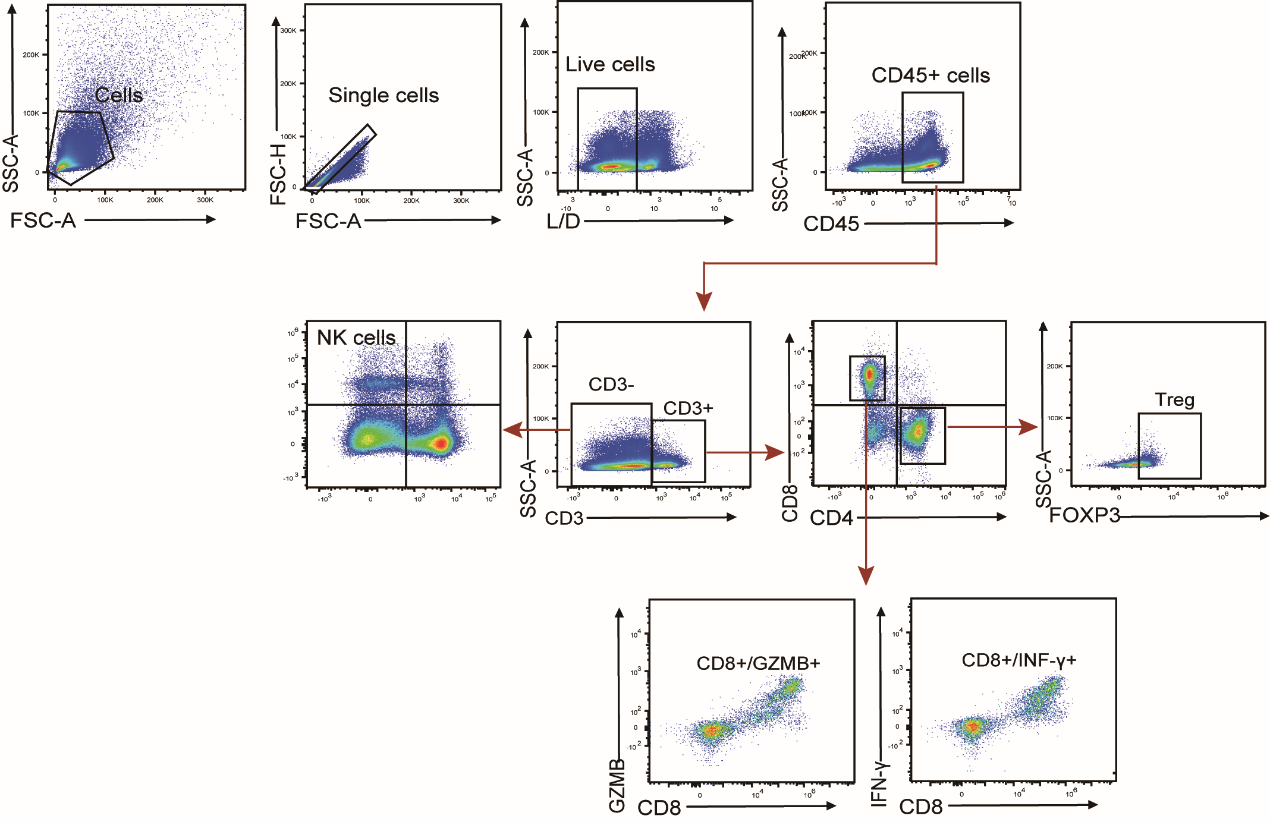


**Figure S9** Gating strategy for flow cytometry analysis of lymphoid and myeloid population in AKR tumors

Table S1 Primer sequences of genes in qRT -PCR assay.

| **Gene** | **Forward Primer** | **Reversed Primer** |
| --- | --- | --- |
| ACTB | CATTGGTAGAAGCGTCGTGAGAGG | GCGGATGTCCACGTCACACTTC |
| ALDOA | AGTTCAGACGAGCCACATTCATTCC | AGTTCAGACGAGCCACATTCATTCC |
| ESRRG | TAGATGCGGAGAACAGCCCA | GGGACAGTAGGGTCAGGCAT |
| ENO1 | GCGGATGTCCACGTCACACTTC | GCGGATGTCCACGTCACACTTC |
| GAPDH | AGATCCCTCCAAAATCAAGTGG | GGCAGAGATGATGACCCTTTT |
| GPI | ACACCAGCAACATTCATTCCACTCC | CATTGGTAGAAGCGTCGTGAGAGG |
| LDHA | CAGCCCGATTCCGTTACCTAATGG | ACACCAGCAACATTCATTCCACTCC |
| PFKM | ACACCAGCAACATTCATTCCACTCC | ACACCAGCAACATTCATTCCACTCC |
| PKM | GCGGATGTCCACGTCACACTTC | AGTTCAGACGAGCCACATTCATTCC |
